# A general Richards family growth curve fit in European livestock for use in physiologically-based toxicokinetic modelling

**DOI:** 10.1186/s12917-025-04982-8

**Published:** 2025-12-04

**Authors:** David Inauen, Leonie Sophie Lautz, Aalbert Jan Hendriks, Ronette Gehring

**Affiliations:** 1 Faculty for Veterinary Medicine, Institute for Risk Assessment Sciences, One Health Pharmacology, Utrecht University, Yalelaan 104-106, Utrecht, 3584 CM The Netherlands; 2 ESQlabs GmbH, Am Sportplatz 7, Saterland, 26683 Germany; 3 Department of Environmental Science, Radboud University Nijmegen, Heyendaalseweg 135, Nijmegen, 6525 AJ The Netherlands

**Keywords:** Beef cattle performance, Dairy cattle performance, Chicken performance, Sheep performance, Growth model, Von Bertalanffy, Gompertz, Physiologically-based modelling, Toxicokinetic

## Abstract

**Background:**

Physiologically-based toxicokinetic models in livestock need to account for growth during long-term exposure studies due to increase in tissue volume and subsequent dilution of concentration. Growth of an average individual of a livestock species was modelled through the Richards family of sigmoidal growth curve models. Average weight gain data of multiple breeds stemming from beef and dairy cattle, laying hens, broiler chickens, and sheep were obtained from scientific literature and industry. Growth curves including the four-parameter Richards curve possessing a flexible inflection point and the three-parameter West, Von Bertalanffy, Monomolecular, and Gompertz curves were fitted to pooled and breed-specific species data. Regression weights were applied to counteract heteroscedasticity, and correlation between residuals was modelled. Fits were compared with the Akaike and Bayesian information criteria, the root mean squared error, and likelihood-ratio tests. Physiological plausibility of curve estimates was also considered. Averages of breed weight gain were used, thus random effects pertaining to individuals were not modelled.

**Results:**

For the pooled fits in beef and dairy cattle, best results were attained by the Von Bertalanffy followed by the West and Richards curves. In laying hen, best fits were attained by the Gompertz curve, followed by the West curve. In male broiler chickens, the Richards curve alone performed best, while in female broiler chickens, the West curve performed similarly well to the Richards curve. For sheep, the Monomolecular curve fitted best. In the breed-specific fits, the Richards curve outperformed the other curve models in all species except laying hens. No statistical difference was found in estimating a breed-specific or a common inflection point of the Richards curve in beef and dairy cattle, provided all other curve parameters were breed-specific. A size-scaled comparison using all animal data showed that the Von Bertalanffy curve returned smallest root mean squared error.

**Conclusion:**

In the pooled fits, parsimonious curve models such as the West and Von Bertalanffy curves performed similarly to the more flexible Richards curve. The parameter estimates in this study can be used for risk assessment to integrate growth of an individual in a generic physiologically-based toxicokinetic model.

**Supplementary Information:**

The online version contains supplementary material available at 10.1186/s12917-025-04982-8.

## Background

For economic reasons, the growth ability of livestock is important. Especially for meat utility, growth is a deciding factor, since it determines feeding amount and length of fattening [1, 2]. For dairy species, growth performance may affect reproduction and have long-term effects on milk yield up to third lactation [3, 4]. Also, the poultry sector changed a lot during the last 50 years, focusing on intensive production systems, and achieving a faster growth rate and slaughter within 42 days [5, 6].

Long-term exposure to toxicological substances may lead to accumulation in plasma, muscle, edible organs, and fat tissue. Growth leads to increased volumes in those bodily compartments, consequently also to dilution of the toxicological concentration. Physiologically based kinetic (PBK) models can account for this factor to give accurate predictions. For animal and environmental health purposes, PBK models including growth curves were previously developed for various fish species [7] or humans [8]. Growth can be provided as a closed-form function, provided the shape and parameters of the growth curve are known.

A growth function should summarise the information provided by observations into a small set of parameters with biological meaning. The Richards family of sigmoidal functions [9], governed by a shape parameter *d*, is commonly used for fitting to growth data. From the Richards curve model, for special values of *d*, named models can be recovered, such as the West [10], Von Bertalanffy [11], Monomolecular [12], Gompertz [13] or Logistic [14] growth curves. In the past, these growth models were applied to evaluate the growth of a wide range of animal species, including birds, mammals, fish, reptiles, and amphibians [15–18]. Such models were usually fitted to individual breeds of a species, and thus represented growth only in the context of a single breed.

The aim of this study was to obtain a general curve fit modelling growth of an individual of livestock species, intended for integration of growth in PBK models. Growth data, specifically weight gain data from different breeds of various common livestock species in Europe, such as beef and dairy cattle, laying hens, broiler chickens, and sheep, were collected from literature. Richards family growth curves were fit to the pooled data of a species to obtain a general curve fit. The range of data and statistical comparison between different curves across multiple species enabled choice of the best-performing curve according to statistical metrics. The performances of the different fits were evaluated using different goodness of fit statistics such as the Akaike information criterion (AIC), Bayesian information criterion (BIC) and the root mean squared error (RMSE), and likelihood-ratio (LR) tests for nested models were applied. Additionally, breed-specific fits were obtained. Finally, a universal comparison between curves was presented using size-scaled animal data.

## Methods

### Data

Growth data of various breeds in beef and dairy cattle, laying hens, broiler chickens and sheep were collected by searching Google and Google Scholar. Search terms for cattle were ‘growth curves’, ‘body weight gain’, ‘beef cattle’, ‘dairy cattle’, and in some cases included breed-specific names: ‘holstein’ and ’angus’. Search terms for laying hens and broiler chickens were ‘performance’ with breed-specific names. Search terms for sheep were ‘growth curves’, ‘body weight gain’ and ‘sheep’.

The mean body weight (BW) data in kg of different breeds were extracted from tables or from growth scatterplots using WebPlotDigitizer [19]. Weekly and monthly time points were converted to daily time points by multiplying by factors 7 and 30.4, respectively. The data search was non-exhaustive, thus not every breed of a species could be represented. Studies with fewer than six data points were excluded. A criterion for inclusion of a dataset was that the first record was close to or at time of birth, such that initial growth lag could be observed. An exception to this were Simmental beef cattle data with records starting at 100 days. Beef cattle data of ten breeds and dairy cattle data of seven breeds were obtained from scientific literature; none of those datasets contained records up to maturity, except for Holstein Friesian (HF) dairy cattle. Subsequently, dairy cattle data were truncated at 800 days to avoid bias by a single breed during later time points. Beef cattle data were male, female or mixed sex, while dairy cattle data were female only. Jersey dairy cattle was fitted separately as it differed strongly from the other breeds [20]. Laying hen data of 21 breeds were obtained directly from optimal rearing performance sheets as laid out by the producers of the breed; an exception was the White Leghorn dataset, which was the composite of multiple publications [21–24]. Similarly, broiler chickens data of six breeds were obtained from producers’ optimal performance data; for each breed, male and female data could be obtained. In contrast to laying hens, broiler chickens data were not recorded up to maturity. Data of seven sheep breeds were obtained from scientific literature; focus was put on sheep reared in Europe, as most of sheep food products consumed in Europe hailed from European breeds [25]. Male, female, and mixed sex data were used. Sheep data were truncated at 500 days due to similar reasoning given for dairy cattle. Data of each species was unbalanced, that is, breed data of a species did not necessarily have the same amounts of observed data available, and time points of observations differed between breeds. An overview of the data structure is given in Table 1. The raw data were provided in “Additional file 2” (AF 2).

**Table 1:** Overview on the datasets of each species

Species	*n* data	*n* breeds	Time span (days)	Range BW (kg)	References
Beef cattle	231	10	0-912	33.1-727.12	[26–31]
Dairy cattle	133	6	0-790	43.0-545.8	[20, 32–34]
Laying hens	1978	20	1-700	0.03–2.22	[35–54]
Broiler chickens	796	6	0–70	0.04–5.79	[55–60]
Sheep	241	7	0-495	3.33–96.5	[61–65]

Number of data points, number of breeds, the time span covered and the range of BW are given. Jersey dairy cattle and White Leghorn laying hen data were not included in this table

### Growth models

For W(t) the BW dependent on time *t* and W’(t) its derivative, the instantaneous relative growth rate (RGR) was given as $\:{\text{W}}^{{\prime\:}}\left(\text{t}\right)/\text{W}\left(\text{t}\right)$. The RGR allowed for growth rate comparison across species of different body size magnitudes. The body weight curve was modelled with the Richards family of sigmoidal curves, using the unified parametrization derived in [66]. The equation of the four-parameter unified Richards growth curve was given as

$$\:\text{W}\left(\text{t}\right)=\text{A}{\left(1+\left({\left({\text{W}}_{0}/\text{A}\right)}^{\left(1-\text{d}\right)}-1\right)\text{exp}\left(-\text{k}\text{U}\text{*}\text{t}/{\text{d}}^{\text{d}/\left(1-\text{d}\right)}\right)\right)}^{1/\left(1-\text{d}\right)}$$

This formulation was useful as each of the four parameters controlled a different aspect of the curve. *A* represented the upper asymptote of the curve (adult weight in kg), *W*_*0*_ the value at time 0 (birth weight in kg) and *kU* the slope at the inflection point (maximal RGR in days^−1^). The parameter *d* ≥ 0 determined the relative height (location) of the inflection point with regard to the upper asymptote, that is, the proportion of weight at maximal RGR to adult weight. The location of the inflection point was a monotonic increasing function given as $\:\text{d}\mapsto\:{\text{d}}^{1/\left(1-\text{d}\right)}$, which determined the ratio of the acceleration phase (initial growth) to the plateauing phase.

A comprehensive overview of growth models including the Richards family was given in Thornley and France 2007 [15]. The underlying biological considerations for the growth models presented involved a closed two-compartmental system, where material was irreversibly transferred from substrate (feed) into a compartment representing BW of animals. Limited substrate led to an upper asymptote of a curve. Inclusion of additional assumptions led to named models of the Richards family; commonly used ones were listed in Table 2. The Richards curve itself was an empirical construct without biological interpretation, but useful due to its flexibility. The growth rate was assumed to be the difference between anabolic and catabolic processes, with anabolism proportional to W(t)^d and catabolism proportional to W(t). For d = 3/4 and d = 2/3, the West and Von Bertalanffy curves were recovered, respectively, see Discussion. Growth rate for the Monomolecular curve (d = 0) was proportional to substrate level $\:\text{A}-\text{W}\left(\text{t}\right),$ up to a rate constant; the curve was non-sigmoidal possessing no inflection point and the maximal RGR was found at the beginning of the curve when t = 0. For the Gompertz curve, the additional assumptions were that substrate level was always non-limiting and that growth rate diminished with increasing senescence, that is, W’(t) was proportional to $\:\text{ln}\left(\text{A}/\text{W}\left(\text{t}\right)\right)\text{*}\text{W}\left(\text{t}\right)$, thus approaching zero while W(t) approached A. The Gompertz curve was a limit case of the Richards curve [67] with d →1. Another important member of the Richards family was the Logistic curve (d = 2) [14], which was not considered in this study.

**Table 2:** Named models and their inflection points

Curve	d	Inflection %A	Weight W(t)	Growth rate W’(t)	Reference
Richards	≥ 0, ≠ 1	0-100%	$\:\text{A}{\left(1+\left({\left({\text{W}}_{0}/\text{A}\right)}^{\left(1-\text{d}\right)}-1\right)\text{exp}\left(-\text{k}\text{U}\text{*}\text{t}/{\text{d}}^{\frac{\text{d}}{1-\text{d}}}\right)\right)}^{1/\left(1-\text{d}\right)}$	$\:\frac{\text{k}\text{U}}{\left(1-\text{d}\right){\text{d}}^{\frac{\text{d}}{1-\text{d}}}}({A}^{1-d}\text{W}{\left(\text{t}\right)}^{\text{d}}-W\left(t\right))$	[9, 66]
West	3/4 (0.75)	31.6%	$\:\text{A}{\left(1+\left({\left({\text{W}}_{0}/\text{A}\right)}^{1/4}-1\right)\text{exp}\left(-64/27\text{k}\text{U}\text{*}\text{t}\right)\right)}^{4}$	$\:\frac{256}{27}\text{k}\text{U}({\text{A}}^{1/4}\text{W}{\left(\text{t}\right)}^{3/4}-\text{W}\left(\text{t}\right))$	[10]
Von Bertalanffy	2/3 (0.67)	29.6%	$\:\text{A}{\left(1+\left({\left({\text{W}}_{0}/\text{A}\right)}^{1/3}-1\right)\text{exp}\left(-9/4\text{k}\text{U}\text{*}\text{t}\right)\right)}^{3}$	$\:\frac{27}{4}\text{k}\text{U}({\text{A}}^{1/3}\text{W}{\left(\text{t}\right)}^{2/3}-\text{W}\left(\text{t}\right))$	[11]
Monomolecular	0	No inflection	$\:\text{A}+\left({\text{W}}_{0}-\text{A}\right)\text{exp}\left(-\text{k}\text{U}\text{*}\text{t}\right)$	$\:\text{k}\text{U}(\text{A}-\text{W}\left(\text{t}\right))$	[12]
Gompertz	→1	36.8%	$\:\text{A}{\left({\text{W}}_{0}/\text{A}\right)}^{\text{exp}\left(-\text{e}\text{*}\text{k}\text{U}\text{*}\text{t}\right)}$	$\:\text{e}\text{*}\text{k}\text{U}\text{*}\text{W}\left(\text{t}\right)\text{ln}\left(\text{A}/\text{W}\left(\text{t}\right)\right)$	[13, 66]

*W(t)* Body weight growth function dependent on time, *W*’*(t)* Rate of growth function, *A* Mature weight (kg), *W*_0_ Birth weight (kg), *kU* Maximal relative growth rate (day^−1^), *d* Richards curve shape parameter

### Fitting

The Richards, West, Von Bertalanffy, Monomolecular and Gompertz curves were fitted to the growth data of beef cattle, dairy cattle, laying hens, broiler chickens and sheep. Curve fitting was conducted with an extended least squares (ELS) nonlinear regression model [68], an extension of weighted-least squares (WLS). The model allowed for (i) heteroscedasticity in the residuals and (ii) correlation between error terms. This could be accounted for by (i) assuming a variance structure on the error term of the regression, leading to a weighted regression, and (ii) assuming a correlation structure between the errors. Further, as a two-stage regression model, it was possible to let growth curve parameters vary by groups, for example breed or sex.

The general curve fit to an animal species was a pooled model, that is, no grouping by breed was applied and a single fixed effect was estimated for each of the curve parameters. This was referred to as Rich_pool_, West_pool_, Bert_pool_, Mono_pool_ and Gomp_pool_, depending on the curves. While biologically there exist obvious differences between breeds, the aim was to obtain a curve fit that described a general breed-agnostic individual of the species. For broiler chickens, the curve parameters and weight function parameter $\:\theta\:$ were additionally stratified by sex. As separate data were available for each sex in Konya Merino and Karagouniko sheep, data for those breeds were split into male and female. To correct for heteroscedasticity in the residuals, weights were applied by assuming a variance structure on the error term of the regression. The variance structure was modelled with a power function $\:\text{g}\left(\widehat{\text{W}}\left({\text{t}}_{\text{j}}\right),{\uptheta\:}\right)=|\widehat{\text{W}}\left({\text{t}}_{\text{j}}\right){|}^{{\uptheta\:}}$ with t_j_ and $\:\widehat{\text{W}}\left({\text{t}}_{\text{j}}\right)$ time point and fitted value of the *j*-th observation of the unweighted regression, respectively. The power function was chosen among a number of candidate functions as it returned the lowest RMSE. Unlike WLS, where the weights are predetermined before fitting, ELS estimated the parameter $\:\theta\:$ during the fitting procedure. The weightings for observations then became $\:1/\text{g}\left(\widehat{\text{W}}\left({\text{t}}_{\text{j}}\right),{\uptheta\:}\right)$. For positive $\:\theta\:$, lower BW fitted values achieved higher weighting; in those cases, more importance was given to datapoints at the beginning stage of growth, where BW was not as dispersed compared to later stages. Since each data set was longitudinal with repeated measures from the same population, residuals stemming from the same breed were correlated. A continuous auto-regressive order 1 (CAR1) [69] correlation structure for each breed was modelled, that is, correlation between residuals from the same breeds was assumed as $\:{{\upvarphi\:}}^{\left|{\text{t}}_{\text{j}}-{\text{t}}_{{\text{j}}^{{\prime\:}}}\:\right|}$, with φ an estimated correlation parameter. The time-continuous nature of the correlation structure could account for unevenly spaced time intervals for each breed. The detailed model specification could be found in AF 1.

In a breed-specific fit, fixed effects for the parameters *A*, *W*_*0*_ and *kU* were calculated per breed (West_breed_, Bert_breed_, Mono_breed_, Gomp_breed_), and for the Richards curve, *d* was also varied by breed (Rich_breed_). This was equivalent to fitting to each breed separately. The resulting fits were compared to a Richards curve fit which also varied *A*, *W*_*0*_ and *kU* by breed, but estimated a single fixed effect for *d* shared by all breeds (Rich_d_). This was to study if models with fixed inflection points were sufficient for breed-specific fits and if there existed statistical improvements in letting the inflection points of the Richards curve vary by breed. Variance and correlation structures were not modelled for the breed-specific fits. Data for Jersey dairy cattle and for White Leghorn laying hens were fitted separately from the pooled and breed-specific models.

The fitting procedure was conducted with the *gnls* function of the *R* package *nlme* v3.1-163, estimating fixed effects via maximum likelihood [70, 71]. Fixed effects estimators are consistent for unbalanced longitudinal data, given sufficient data [72]. Starting values were calculated by fitting the *nls* function in *R* to each breed separately and then either providing the mean of the estimated parameters (pooled model) or the parameter estimates of each individual breed (breed-specific model). For the Gomp_breed_ fit in sheep, the estimates of the Bert_breed_ fit were used as initial values. In the pooled model, the variance structure was given by the variance function ‘varPower’ and the correlation structure was given by the correlation function ‘corCAR1’. To ensure a fit to species data, the ‘nlsTol’ parameter in the *gnls* function was increased for a select few species.

### Evaluation

For both the pooled and the breed-specific models, the AIC, BIC and RMSE were chosen as evaluation criteria. The AIC penalized models based on their number of parameters, while the BIC in addition took the sample size into account. The detailed formulas for each criterion and the RMSE can be found in AF 1. Fits with lowest AIC, BIC and RMSE that also exhibited plausible estimates were considered optimal. Biological plausibility was considered for some outlier parameter estimates: Estimates for *W0* were considered plausible if the value was in range of the initial observations of the datasets. The *A* estimates were compared with breed-specific literature data to evaluate plausibility. For nested models, LR tests were applied at a significance level of 0.05. Specifically, in the pooled fit, the Richards curve was compared to the other named models; in the breed-specific fit, Rich_d_ was tested against the other named models, including Rich_breed_. Homo/heteroscedasticity, normality in the residuals and correlation between errors was detected by visual inspection of fitted vs. normalized residual plots, quantile-quantile plots and temporal semivariograms, respectively.

### Universal comparison

West et al. 2001 [10] suggested a universal dimensionless curve transforming species data onto the same scale such that growth of multiple species was modelled by a single curve. A similar comparison between the curves was implemented here, scaling all species' BW data by the adult weight obtained from the pooled fits. From the Richards curve equation, it can be seen that BW divided by *A* followed a dimensionless curve $\:{\uptau\:}\mapsto\:{\left(1-{e}^{-\tau\:}\right)}^{\frac{1}{1-d}},\:0\le\:\text{d}<1$ where

$$\:{\uptau\:}\equiv\:\frac{\text{ku*t}}{{\text{d}}^{\frac{\text{d}}{1-\text{d}}}}-\text{ln}\left(1-{\left(\frac{{\text{W}}_{0}}{\text{A}}\right)}^{1-\text{d}}\right)\:$$

and for the Gompertz curve, $\:\tau\:\mapsto\:1-{e}^{-\tau\:}$ where

$$\:{\uptau\:}\equiv\:-\text{ln}\left(1-{\left(\frac{{\text{W}}_{0}}{\text{A}}\right)}^{\text{exp}\left(-\text{e}\text{*}\text{k}\text{U}\text{*}\text{t}\right)}\right),\:$$

enabling comparison of curves on the aggregate of species data using the RMSE.

## Results

### A general breed-agnostic curve for each species

The fits to the pooled data are presented in Table 3.

**Table 3:** Best fits pooled model for each species and curve

Species	Fit	A (kg)	W_0_ (kg)	kU (10^−3^ day^−1^)	d	σ	θ	AIC	BIC	RMSE
Beef cattle	Rich_pool_	710.1 ± 51.6	38.5 ± 9.2	1.4 ± 0.1	0.69 ± 0.15	12.6	0.23	1777.8	1801.9	41.9
	West_pool_	695.4 ± 33.9	40.2 ± 8.2	1.4 ± 0.1	0.75 (fixed)	12.3	0.23	1776.1	1796.8	41.9
	Bert_pool_	714.5 ± 37.0	37.9 ± 8.3	1.4 ± 0.1	0.67 (fixed)	12.5	0.23	1776	1796.6	41.8
	Mono_pool_*	1472.7 ± 341.0	46.6 ± 10.2	0.7 ± 0.2	0 (fixed)	11.8	0.26	1799.5	1820.2	45.4
	Gomp_pool_	658.0 ± 28.5	47.9 ± 8.2	1.5 ± 0.1	d↘1 (fixed)	12.6	0.22	1779.3	1799.9	42.5
Dairy cattle	Rich_pool_	743.7 ± 96.8	41.5 ± 2.7	1.0 ± 0.1	0.48 ± 0.16	0.5	0.64	998.4	1018.6	20.4
	West_pool_	641.6 ± 27.8	43.4 ± 2.6	1.2 ± 0.1	0.75 (fixed)	0.6	0.61	1000	1017.3	21.1
	Bert_pool_	665.0 ± 31.3	42.7 ± 2.6	1.2 ± 0.1	0.67 (fixed)	0.5	0.63	998.2	1015.6	20.9
	Mono_pool_*	1808.9 ± 566.9	39.7 ± 3.1	0.4 ± 0.2	0 (fixed)	0.7	0.59	1006.1	1023.5	20.9
	Gomp_pool_*	595.9 ± 21.7	45.8 ± 3.0	1.4 ± 0.1	d↘1 (fixed)	0.8	0.56	1007.6	1024.9	21.9
Laying hen	Rich_pool_	1.99 ± 0.01	0.39 ± 0.02	5.7 ± 0.1	2.69 ± 0.14	0.27	−0.63	−14545.6	−14506.5	0.22
	West_pool_*	1.91 ± 0.01	0.03 ± 0^+^	7.4 ± 0.1	0.75 (fixed)	0.16	−0.26	−14178.6	−14145.1	0.16
	Bert_pool_*	1.91 ± 0.01	0.01 ± 0^+^	7.5 ± 0.1	0.67 (fixed)	0.16	−0.28	−14,094	−14060.5	0.16
	Mono_pool_*	1.94 ± 0.01	−0.67 ± 0.04	13.6 ± 0.2	0 (fixed)	0.61	−2.30	−12,835	−12801.5	0.18
	Gomp_pool_*	1.92 ± 0.01	0.08 ± 0^+^	7.2 ± 0.1	d↘1 (fixed)	0.16	−0.28	−14339.7	−14306.2	0.16
Broiler M	Rich_pool_	8.40 ± 0.38	0.05 ± 0^+^	12.1 ± 0.3	0.80 ± 0.01	0.2	0.68	−2773.7	−2745.8	0.52
	West_pool_*	8.28 ± 0.31	0.03 ± 0.1	11.8 ± 0.2	0.75 (fixed)	0.2	0.59	−2715.5	−2691.5	0.48
	Bert_pool_*	9.03 ± 0.25	0.02 ± 0^+^	10.7 ± 0.2	0.67 (fixed)	0.3	0.41	−2555.5	−2531.6	0.48
	Mono_pool_	NA	NA	NA	0 (fixed)	NA	NA	NA	NA	NA
	Gomp_pool_*	5.06 ± 0.39	0.04 ± 0^+^	20.7 ± 0.3	d↘1 (fixed)	0.2	1.11	−2001.4	−1981.4	0.52
Broiler F	Rich_pool_	6.76 ± 0.33	0.06 ± 0.1	12.2 ± 0.3	0.77 ± 0.01	0.2	0.66	−2846	−2818.1	0.47
	West_pool_	6.66 ± 0.33	0.04 ± 0.01	12.3 ± 0.1	0.75 (fixed)	0.29	0.67	−2846.4	−2822.5	0.42
	Bert_pool_*	6.97 ± 0.22	0.02 ± 0.01	11.3 ± 0.2	0.67 (fixed)	0.3	0.46	−2682.8	−2658.9	0.42
	Mono_pool_	NA	NA	NA	0 (fixed)	NA	NA	NA	NA	NA
	Gomp_pool_*	3.97 ± 0.37	0.05 ± 0^+^	19.5 ± 0.4	d↘1 (fixed)	0.18	1.13	−1900	−1880.1	0.41
Sheep	Rich_pool_	NA	NA	NA	NA	NA	NA	NA	NA	NA
	West_pool_	62.6 ± 4.0	5.7 ± 1.1	4.1 ± 0.3	0.75 (fixed)	1.6	0.41	647.5	667.9	5.8
	Bert_pool_	64.0 ± 4.1	5.4 ± 1.1	3.9 ± 0.3	0.67 (fixed)	1.6	0.40	643.8	664.2	5.6
	Mono_pool_	88.0 ± 10.0	4.0 ± 0.9	3.0 ± 0.5	0 (fixed)	1.6	0.35	619.5	639.9	4.6
	Gomp_pool_	59.0 ± 4.1	6.7 ± 1.2	4.7 ± 0.3	d↘1 (fixed)	1.6	0.45	658.4	678.8	6.6

The parameter estimates are provided with the standard errors. Instances where no convergence was achieved are indicated by “NA”. 0^+^ indicates positive values smaller than 0.01

*A* Mature weight, *W*_0_ Birth weight, *kU* Maximal relative growth rate, *d* Richards curve shape parameter, *σ* Residual standard error, *θ* Parameter for variance function, *AIC* Akaike information criterion, *BIC* Bayes information criterion, *RMSE* Root mean squared error

*Fit significantly different than Rich_pool_

#### Beef cattle

**Fig. 1:**
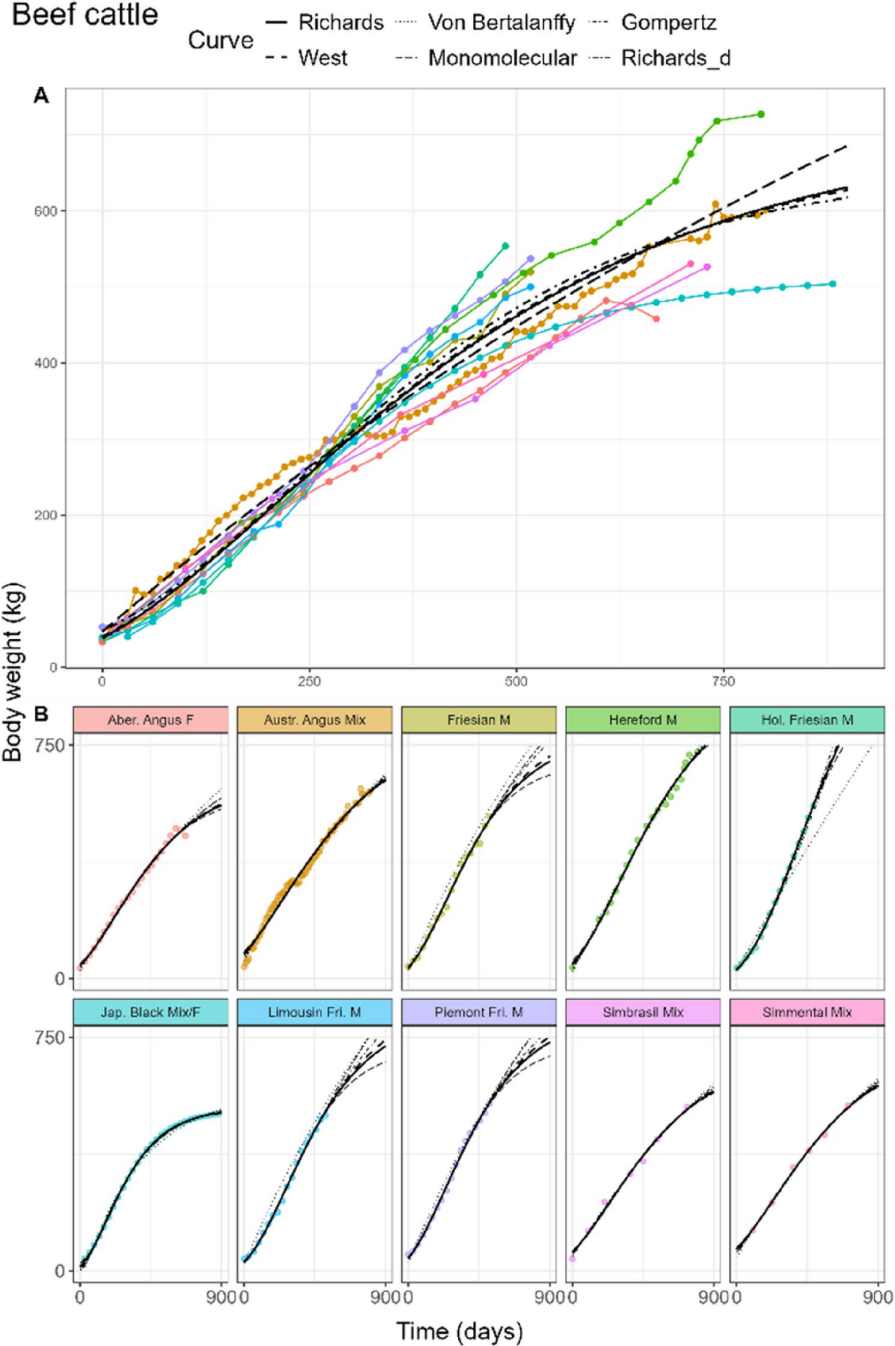
General (**A**) and breed-specific (**B**) growth curve fits in beef cattle of the Richards (solid), West (dashed), Von Bertalanffy (dotted), Monomolecular (longdash), and Gompertz (dotdash) curves. Richards curve with common d parameter (twodash) is shown only in the breed-specific plots. Sex is indicated next to each breed as male (M), female (F), or mixed sex (Mix)

The fitted curves for beef cattle of the pooled model can be found in Fig. 1A. The lowest AIC and BIC in beef cattle were attained by the Von Bertalanffy curve, followed by the West curve (Table 3). RMSE was similar among the Richards, West and Von Bertalanffy fits. No significant differences between the Richards and the West, Von Bertalanffy and Gompertz fits were found. The Richards curve estimated *d* at 0.69 (30.2% of adult weight), returning similar estimates as the Von Bertalanffy curve. Adult weights were estimated in the range of 658.0 to 714.5 kg, with the exception of the Monomolecular curve estimating 1472.7 kg. Birth weights varied between 37.9 and 47.9 kg, while estimates of *kU* were fairly similar for all curves at 0.0014–0.0015 per day, except for the Monomolecular curve with 0.0007 per day.

#### Dairy cattle

**Fig. 2:**
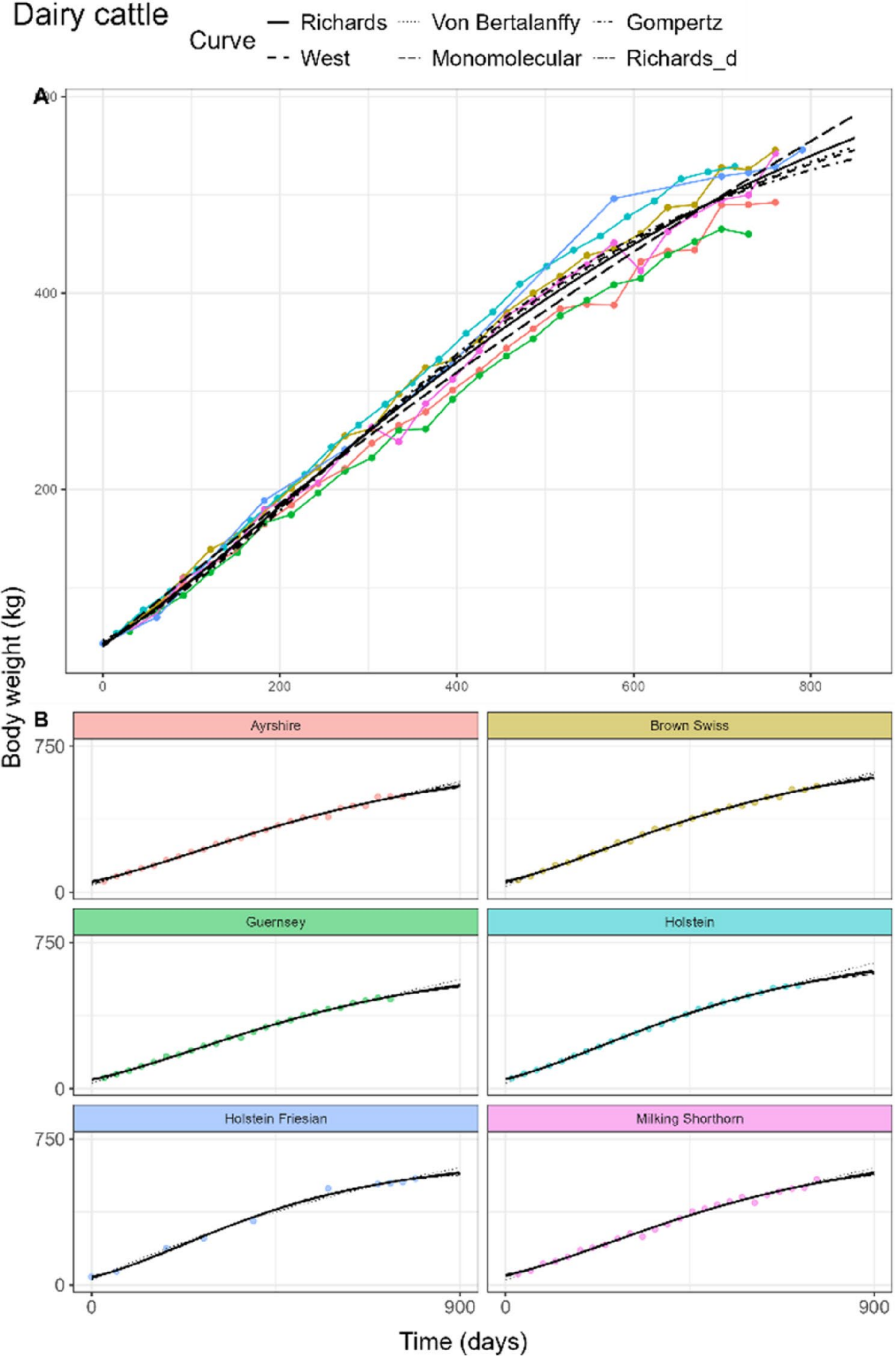
General (**A**) and breed-specific (**B**) growth curve fits in dairy cattle of the Richards (solid), West (dashed), Von Bertalanffy (dotted), Monomolecular (longdash), and Gompertz (dotdash) curves. Richards curve with common d parameter (twodash) is shown only in the breed-specific plots

The fitted curves for dairy cattle of the pooled model can be found in Fig. 2A. The lowest AIC and BIC were attained by the Von Bertalanffy curve (Table 3). The lowest RMSE was attained by the Richards curve. The Monomolecular and Gompertz fits differed statistically from the Richards fit. The Richards curve estimated *d* at 0.48, that is, inflection at 24.4% of the adult weight, which was a considerably lower value than in the Richards fit to beef cattle. The adult weight was in general lower than in beef cattle, varying from 595.9 to 743.7 kg, with an extreme prediction of the Monomolecular curve at 1808.9 kg. Estimates of *W*_*0*_ ranged between 39.7 and 45.8 kg, similar to beef cattle, while *kU* was estimated at lower values than in beef cattle, from 0.0004 to 0.0014 per day.

#### Laying hens

**Fig. 3:**
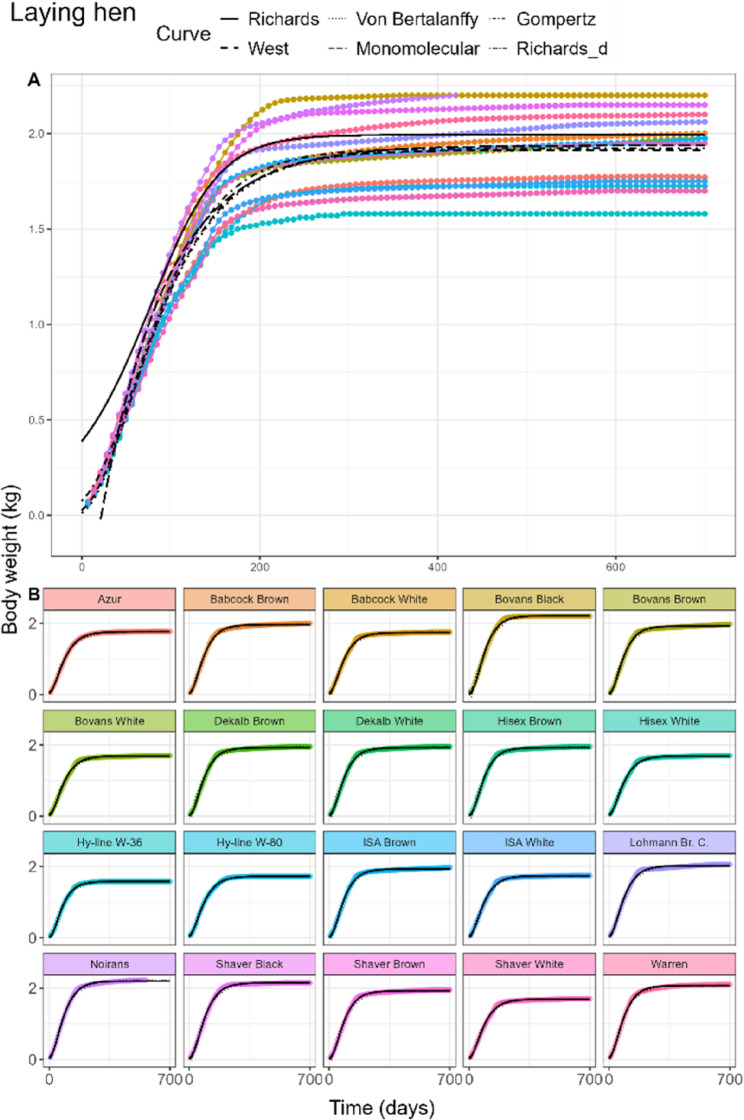
General (**A**) and breed-specific (**B**) growth curve fits in laying hens of the Richards (solid), West (dashed), Von Bertalanffy (dotted), Monomolecular (longdash), and Gompertz (dotdash) curves. Richards curve with common d parameter (twodash) is shown only in the breed-specific plots

The fitted curves for laying hens of the pooled model can be found in Fig. 3A. The Richards fit attained the lowest AIC and BIC (Table 3), but overpredicted birth weight with a value five times higher than the initial mean weight of the data (0.063 kg). *d* was estimated at 2.69, with an inflection point at 55.7% of the adult weight. All other curve fits were statistically different from the Richards fits, with the Gompertz fit attaining lowest AIC and BIC among them. Adult weight estimates ranged between 1.91 and 1.99 kg, while birth weights of the West fit were 0.03 kg, slightly underestimated for the Von Bertalanffy fit (0.01 kg) and slightly overestimated for the Gompertz fit (0.08 kg). The Monomolecular curve predicted a physiologically implausible negative birth weight of −0.67 kg. Estimates of *kU* ranged from 0.0057 to 0.0136 per day. In contrast to the other species, $\:\theta\:$ was estimated negative for all curves, thus more regression weight was given to the later time points.

#### Broiler chickens

**Fig. 4:**
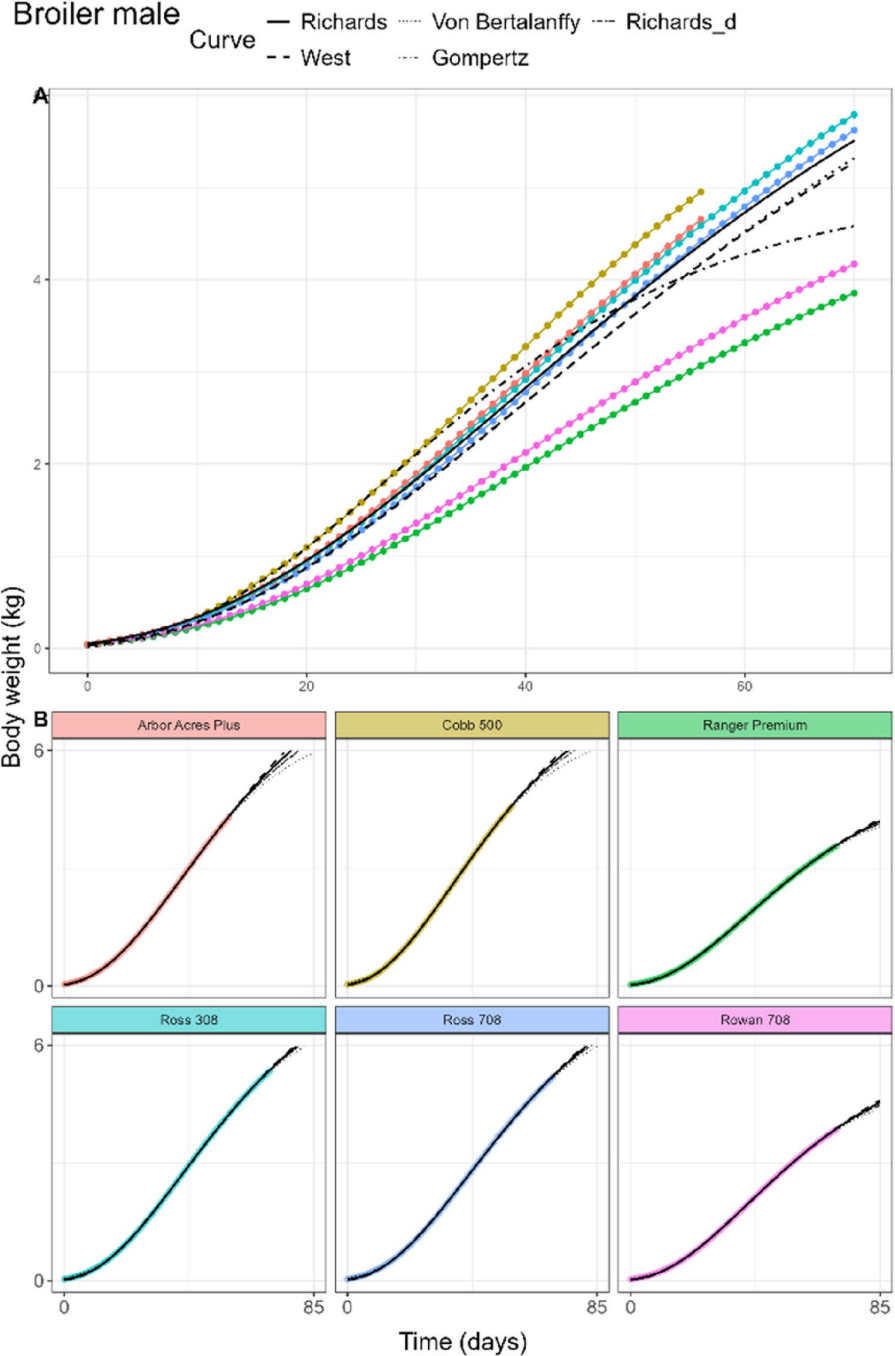
General (**A**) and breed-specific (**B**) growth curve fits in male broiler chickens of the Richards (solid), West (dashed), Von Bertalanffy (dotted), and Gompertz (dotdash) curves. Richards curve with common d parameter (twodash) is shown only in the breed-specific plots

**Fig. 5:**
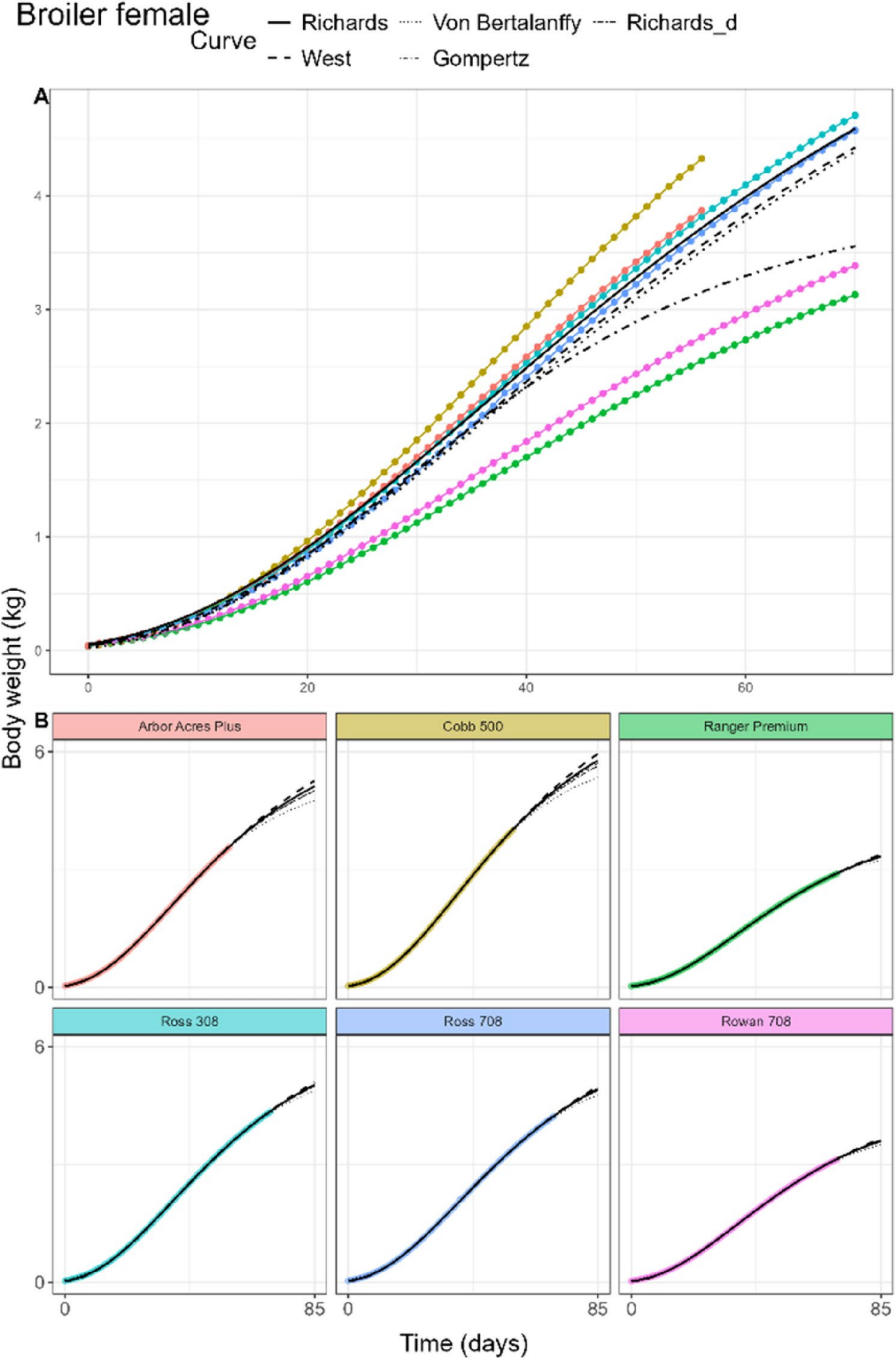
General (**A**) and breed-specific (**B**) growth curve fits in female broiler chickens of the Richards (solid), West (dashed), Von Bertalanffy (dotted), and Gompertz (dotdash) curves. Richards curve with common d parameter (twodash) is shown only in the breed-specific plots

The fitted curves for male broilers and female broilers of the pooled model can be found in Figs. 4A and 5A, respectively. In male boilers, the Richards fit attained lowest AIC and BIC, while in female broilers the same was true for the West fit (Table 3). Lowest RMSE in male broilers was found in the West and Von Bertalanffy fits while in female broilers this was true for the Gompertz fit. In female broilers, the West fit was not significantly different from the Richards fit. Inflection points differed only slightly between sexes, with *d* being estimated at 0.80 (32.8% of adult weight) and 0.77 (32.1% of adult weight) for male and female broilers, respectively. Adult weight estimates ranged between 5.06 and 9.03 kg for male broilers and 3.97 and 6.97 kg for female broilers. Birth weight estimates of each curve were similar in both sexes, ranging between 0.02 and 0.06 kg, with the lowest birth weight given by the Von Bertalanffy fit. *kU* estimates in both sexes were higher than those of laying hens, in a wide range from 0.0107 to 0.0207 per day, with higher values in female broilers. The Monomolecular curve did not achieve any fit on the data, despite adjustments in the tolerance parameters of the fitting procedure.

#### Sheep

**Fig. 6:**
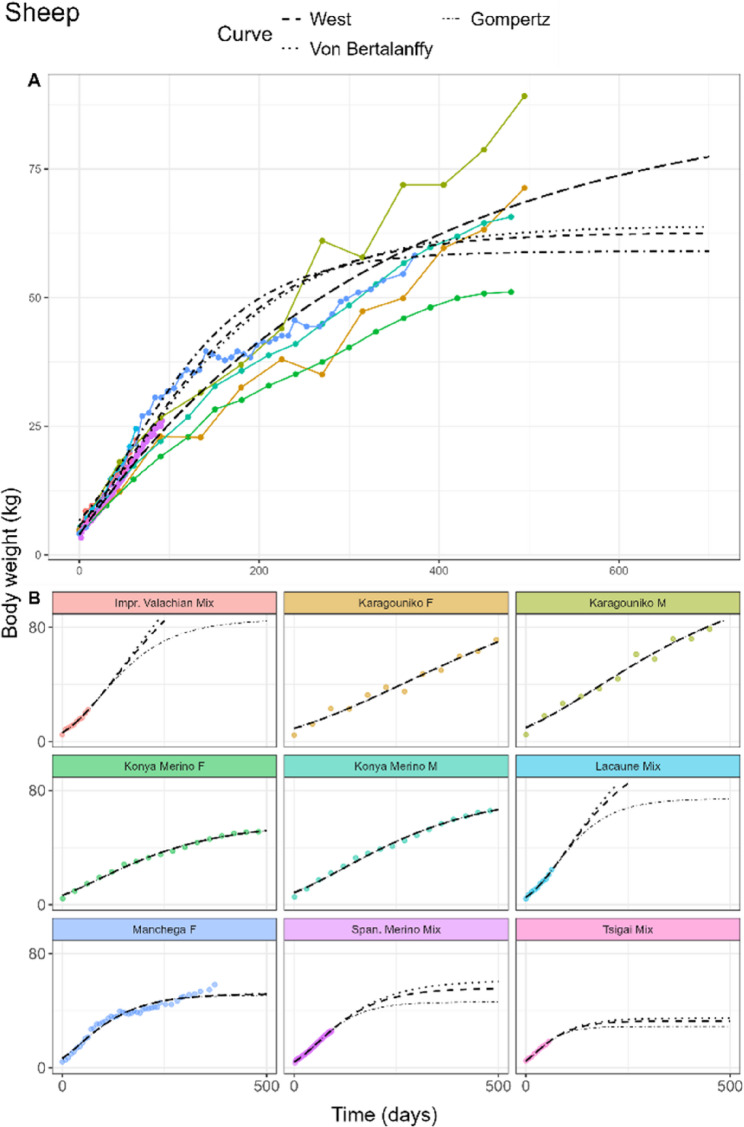
General (**A**) growth curve fits in sheep of the West (dashed), Von Bertalanffy (dotted), Monomolecular (longdash), and Gompertz (dotdash) curves. The breed-specific fit (**B**) only includes the West, Von Bertalanffy, and Gompertz curves. Sex is indicated next to each breed as male (M), female (F), or mixed sex (Mix)

The fitted curves for sheep of the pooled model can be found in Fig. 6A. The Monomolecular curve attained the lowest AIC, BIC, and RMSE (Table 3). The West, Von Bertalanffy, and Gompertz curves estimated A at 62.6, 64.0 and 56.0, respectively, while the Monomolecular curve gave an estimate of 88.0 kg. Sheep birth weights ranged between 4.0 and 6.7 kg, and *kU* values ranged between 0.0030 and 0.0047 per day. The Richards curve did not achieve any fit on the data.

#### Diagnostics (all animals)

LR tests showed that modelling the covariance and correlation structures led to significantly improved fits compared to the unweighted regression. Diagnostic plots are provided in the AF 1 Figs. 1–18. Heteroscedasticity issues of the residuals were adequately resolved in most fits, except in sheep (AF 1 Fig. 16). For each species, outliers in the normalized residuals were found for small fitted values; distributions of residuals appeared to have heavier tails than a Normal distribution. The temporal semivariograms for sheep (AF 1 Fig. 18) revealed that the chosen correlation structure was inadequate for explaining correlations. For all species and curve fits, φ was estimated with a value greater than 0.99.

#### Correlation between parameters

Correlations between *d* and *A* of the pooled Richards fits were strongly negative (< −0.7) in cattle and moderately positive (> 0.2) in chickens. Correlations between *d* and *kU* were strongly positive (> 0.7), in all species except laying hens. Furthermore, moderate to strong positive (> 0.3) correlation between *d* and *W*_*0*_ was found in all species. Adult weight *A* had strong negative correlation (< −0.7) with *kU*, except in broiler chickens. *A* and *W*_*0*_ were not correlated in beef cattle, weakly negatively correlated in dairy cattle, and strongly positively (> 0.7) correlated in chickens. Estimates of *kU* were the lowest in cattle and the highest in chickens, with sheep in-between. Beef cattle and broiler chickens mostly had higher *kU* and *A* than dairy cattle and laying hens, respectively.

### Breed-specific fits with common or varying d parameter

The breed-specific model showed clear improvements in the evaluation metrics over the pooled model, indicating that there were distinct differences between breeds. The Richards curve had a significantly better fit than the other named models in almost all species, with some exceptions, as seen in Table 4. The differences between the Rich_d_ and Rich_breed_ fits were further discussed. BICs and RMSEs are presented in AF 1 Tbls. 1 and 2.

**Table 4:** AICs of curve fits when varying parameters by breed

Species	Rich_d_	d (SE)	West_breed_ (d = 3/4)	Bert_breed_ (d = 2/3)	Mono_breed_ (d = 0)	Gomp_breed_ (d↘1)	Rich_breed_	d (Range)
Beef cattle	1881.4	0.38 ± 0.09	1894.8^‡^	1889.1^‡^	2161.8^‡^	1916.1^‡^	1885.6	0.09–0.56
Dairy cattle	984.2	0.58 ± 0.12	984.3	982.7	1011.8^‡^	993.7^‡^	986.8	0.22–1.15
Laying hen	−9450.4	0.99 ± 0.01	−9183.0^‡^	−8961.2^‡^	−5621.4^‡^	−9451.5	−9533.4*	0.76–1.40
Broilers M	−3176.4	0.84 ± 0^+^	−2517.0^‡^	−2053.6^‡^	NA	−2207.3^‡^	−3322.0*	0.77–0.87
Broilers F	−3328.1	0.82 ± 0^+^	−2922.6^‡^	−2329.5^‡^	NA	−2238.7^‡^	−3663.4*	0.79–0.84
Sheep	NA	NA	930.5	922.6	NA	935.0	NA	NA

Estimates of d were added for the Rich_d_ and Rich_breed_ models. Instances where no convergence was achieved are indicated by “NA”. Dairy cattle and laying hens were without Jersey and White Leghorn breeds, respectively

*d* Richards curve shape parameter

^‡^Fit significantly worse than Rich_d_

*Fit significantly better than Rich_d_

#### Beef and dairy cattle

In beef and dairy cattle, Rich_d_ estimated *d* at 0.38 and 0.58, respectively, corresponding to inflection at 21.0 and 27.3% of the adult weight (Table 4). These estimates reflected the ‘average’ location of the inflection point between breeds of a species. Compare to the breed agnostic Rich_pool_ fits where the respective estimates of *d* were 0.69 and 0.48. Rich_breed_ estimated inflection of individual beef and dairy cattle breeds on ranges 0.7–26.8% and 14.4–39.4%, respectively, indicating that depending on breed and data available, inflection could drastically vary. In dairy cattle, there were no statistical differences between Rich_d_ and the West and Von Bertalanffy fits. Further, there were also no statistical differences between Rich_d_ and Rich_breed_ for both beef and dairy cattle, indicating that estimating a single inflection points was statistically similar as stratifying by breed. The curves of the breed-specific fits for beef and dairy cattle can be found in Figs. 1B and 2B, respectively. Estimated parameter values, including Jersey cattle, can be found in AF 1 Tbls. 3 and 4.

#### Laying hens, broiler chickens and sheep

In laying hens, Rich_d_ estimated *d* at 0.99, compared to the Rich_pool_ estimate of *d* = 2.69. The inflection points of the individual hen breeds in Rich_breed_ were estimated between 31.9% and 43.1% of the adult weight. For some breeds like Bovans Brown, the slow linear growth in the later time points was not characterised by the fits (Fig. 3B). In both male (Fig. 4B) and female broilers (Fig. 5B), only small differences of the *d* estimates between the Rich_pool_ model (M: 0.80, F: 0.77) and Rich_d_ model (M: 0.84, F: 0.82) were found. Rich_breed_ estimated similar ranges of *d* for both sexes. In sheep (Fig. 6B), only the West, Von Bertalanffy and Gompertz models reached convergence. Estimated parameter values, including White Leghorn hen, can be found in AF 1 Tbls. 5–8.

#### Breed parameter estimates and biological plausibility

**Fig. 7:**
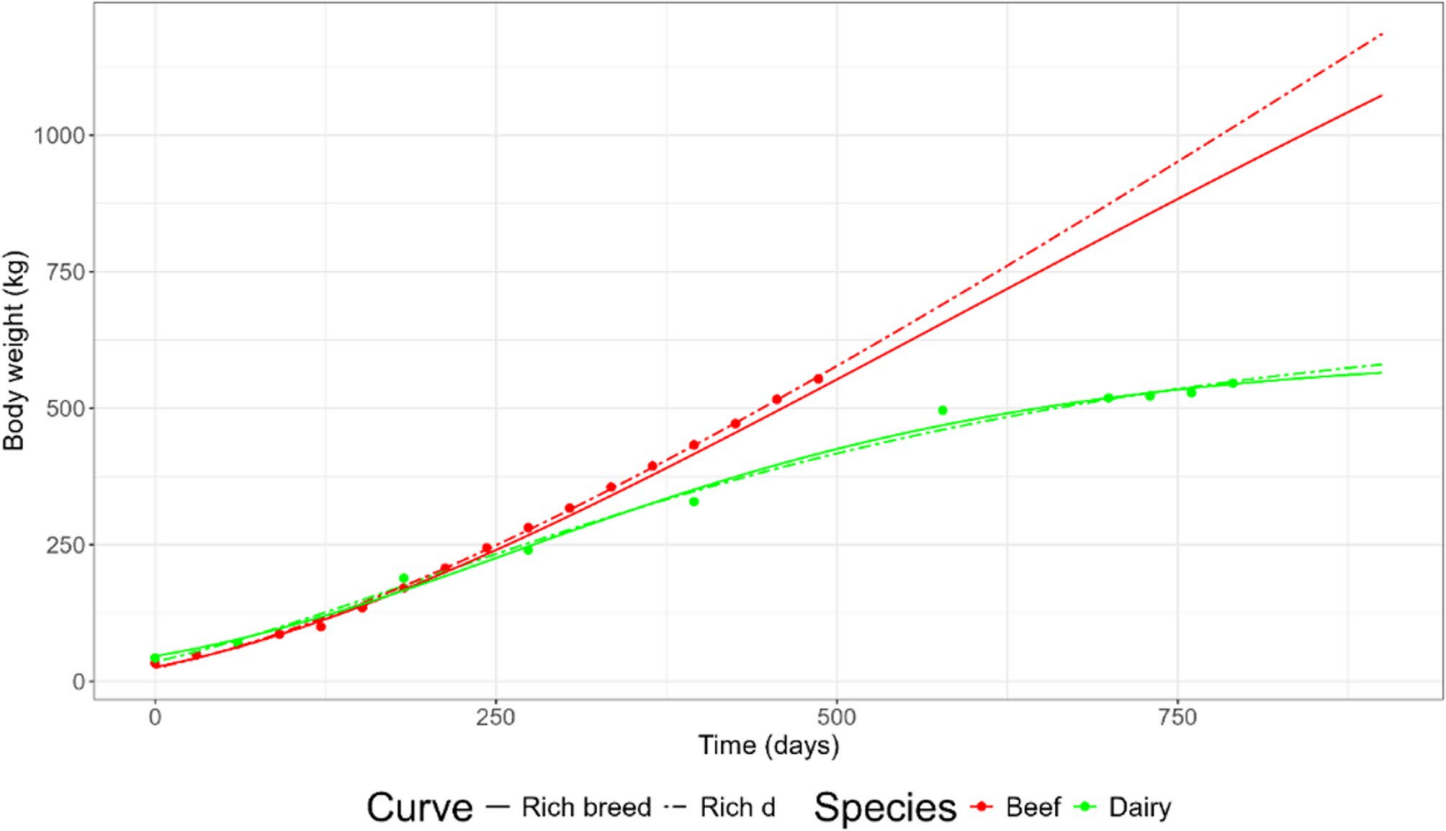
Rich_breed_ (solid) and Rich_d_ (twodash) curve fits to Holstein Friesian beef and dairy cattle data

Physiologically implausible curve fits were returned in a small number of beef cattle and sheep breeds. This was exemplified in male HF beef cattle (Fig. 7). Rich_d_ estimated HF beef cattle adult weight at 6325.2 kg, while Rich_breed_ returned a less extreme but still implausible value of 2662.4 kg and a breed-specific *d* estimate of 0.49. Estimates from other curves such as the von Bertalanffy (1570.2 kg) and Gompertz (1012.5 kg) were lower. In comparison, female HF dairy cattle adult weight estimates of the Rich_d_ and Rich_breed_ models were 675.6 kg and 601.1 kg, respectively, with the latter estimating *d* for HF dairy cattle at 1.15. Actual adult weights for HF cattle range between 1150 and 1200 kg for males and 650–750 kg for females [73]. The HF beef cattle data did not cover enough time points to exhibit a sigmoidal shape due to a lack of records in later time points, in contrast to the HF dairy cattle data.

For sheep, some adult weights were overestimated (Improved Valachian) or underestimated (Tsigai) (AF 1 Tbl. 8). For Karagouniko, all curve fits estimated higher adult weights in female sheep which was at odds with literature values [74]. For comparison between estimated and actual adult weights, literature values of adult weights of cattle and sheep have been added in AF 1 Tbls. 3, 4, and 8. For broilers, sources of reliable adult weight values were not obtainable, while for laying hens, estimates of A can be directly compared to the data.

Diagnostic plots revealed that regression assumptions were not fulfilled on the breed level for some species-curve combinations (not shown). Modelling the variance structure did not alleviate the issues, or led to non-convergence. Heteroscedasticity of residuals may lead to inconsistent parameter estimates in nonlinear regression [75].

### Universal comparison

**Fig. 8:**
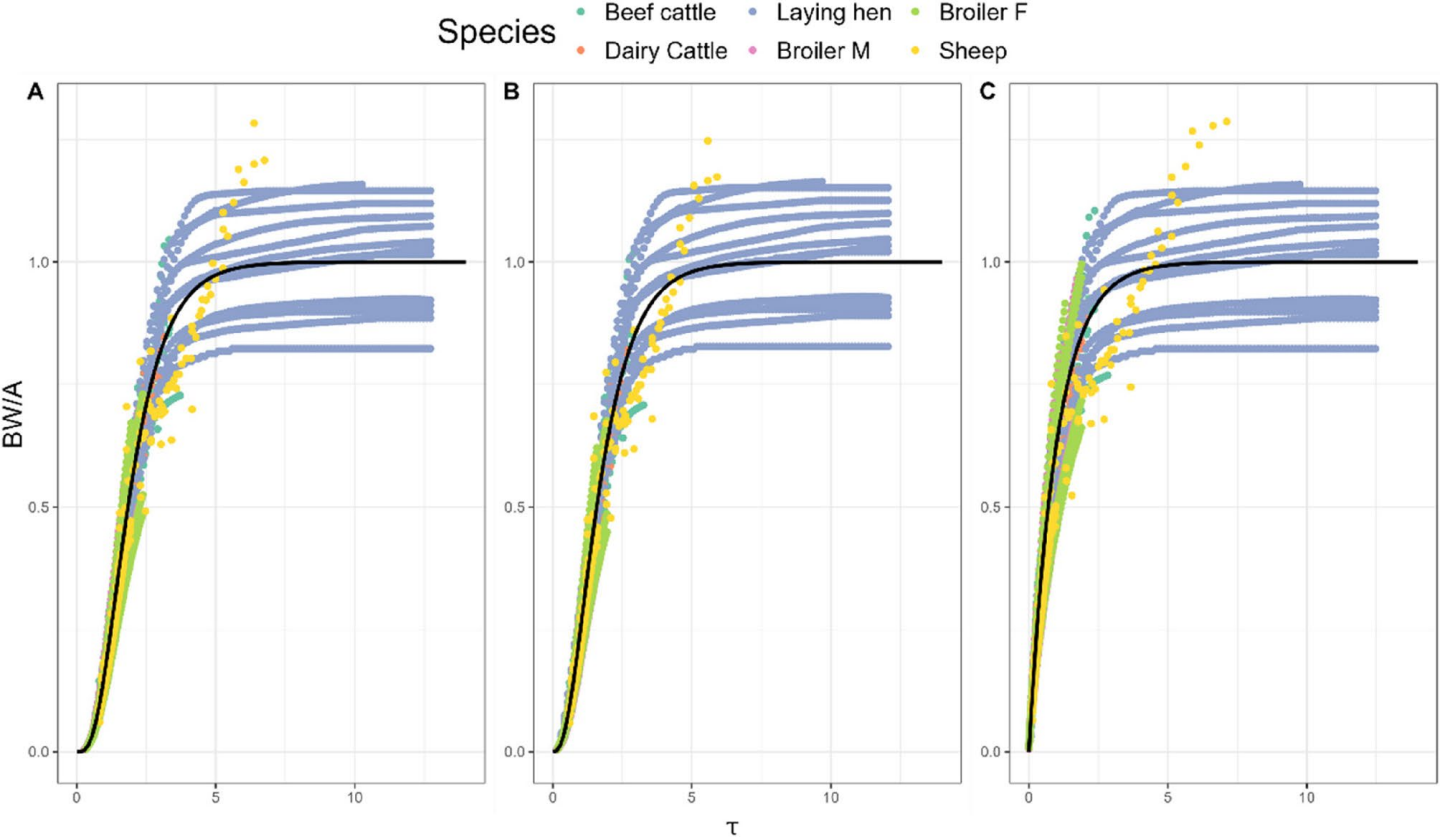
Scaled body weights of aggregated animal data. The solid line depicts the dimensionless curve for the (**A**) West, (**B**) Von Bertalanffy and (**C**) Gompertz curves

Species data were aggregated and scaled using the parameters obtained from the pooled fits. The transformed data and dimensionless curve is plotted in Fig. 8. The RMSEs were 0.076, 0.075 and 0.082 for the West, Von Bertalanffy and Gompertz curves, respectively. Richards and Monomolecular curves were omitted as pooled fits were not achieved for each species.

## Discussion

### Pooled model

Limited statistical differences between the Richards, West, and Von Bertalanffy fits in beef and dairy cattle indicated that a more parsimonious model was equally suited as a general growth curve in place of the more complex Richards curve. For laying hens, the Richards curve may have achieved the best fit, but parameter estimates of the Gompertz or West curves were more plausible. In female broilers, there was little difference between the Richards and West curves, while in male broilers, the Richards curve performed best. Estimates of adult weights varied strongly between the curves, possibly due to the broiler data being cut off before reaching maturity. In sheep, the Monomolecular curve returned the best fit. Evaluation metrics such as information criteria and LR testing could not solely be relied upon in choosing an optimal model; plausibility in the parameters needed to be considered additionally.

### Breed-specific model

Overall, the Richards curve was equally or better suited for breed-specific fits, compared to other named models. For beef and dairy cattle, there was no statistically significant difference between Rich_breed_ and Rich_d_, indicating that breed-specific estimation of inflection points does not lead to significant differences in curve fits, compared to estimating a single inflection point for a species. For a few breeds, lack of sufficient data coverage led to incomplete sigmoidal shapes in the growth curves and consequently to physiologically implausible estimates. In addition, regression assumptions were not fulfilled in some breeds.

The breed-specific fits performed clearly better than the pooled fits, which was expected since from a biological standpoint, it is clear that growth varies between different breeds. If integrated as growth curves into PBK models, there may arise differences in time-concentration curves dependent on whether parameters from the pooled fit or the breed-specific fit are used. The advantage of modelling growth with the pooled fit is that it can generalize for a species and is not as sensitive to variability in data, compared to breed-level data. However, for clear outliers such as Jersey dairy cattle, a breed-specific fit may be more optimal.

### Universal comparison

Taylor 2008 [76] put forward the idea of metabolic age, scaling time by adult BW with an allometric exponent of 0.27, establishing a universal life cycle of a mammal. West et al. 2001 [10] developed a related concept, proposing a universal growth curve for a multitude of animal species, again based on scaling with adult BW, with a similar allometric exponent of 0.25. In the present work, this idea was generalized for comparison between curves across species with varying BWs. It was found that the Von Bertalanffy universal curve returned the lowest RMSE, which is not surprising as the Von Bertalanffy fits performed consistently well in the pooled model.

### Growth curve literature

Both species and breed-specific curve comparisons using the Richards family and other curve models have been employed in literature; however, an overall best curve for describing growth of a species was not obvious. A small number of publications providing data for the present study applied their own growth curve analysis; in those publications curve comparisons were conducted in single breeds alone (AF 1 Tbl. 9). In the present study’s breed-specific model, curves were compared while fitted simultaneously to multiple breeds, thus only indirect comparison to literature is possible.

For the pooled beef cattle fit in our study, the Richards, West and Von Bertalanffy curves returned the best results, while for breed-specific fits, the Richards curve performed best. The Hereford beef cattle study [28] used in this publication found that the Von Bertalanffy and Gompertz curves achieved better fits than the Richards curve. Zimmermann et al. 2019 [77] found that the Brody curve fit best a population of crossbred beef cows, compared to spline and quadratic curves. That study used cattle data starting at 180 days, thus the initial growth phase was not considered.

In dairy cattle, the present study found that the Richards and Von Bertalanffy curves were best suited for a pooled fit, while for breed-specific fits, the West model was an additional suitable candidate. In Perotto et al. 1992 [78], the Richards curve described best growth in three breeds of dairy cattle, among a selection of named models. Berry et al. 2005 [79] chose the Von Bertalanffy over the Monomolecular curve to describe growth in three strains of dairy cattle. Unterauer et al. 2021 [80] fitted the Bertalanffy-Püttner (BP) model, a five-parameter generalization of the Richards curve, to various cattle data. The authors found that the Richards curve underperformed on their data, and a sigmoidal curve with inflection point at 24.6% of adult weight described the most sigmoidal growth datasets in an optimal manner.

An extensive review of use frequency and comparisons of different growth curves in poultry (chicken, turkey, quail, and others) was available [81], where it was found that the majority of growth curves used in poultry literature were the Gompertz and Logistic, followed by the Richards and Von Bertalanffy curves. Studies which compared Richards family growth curves in chickens revealed that the Richards curve generally performed best; if the Richards curve was not included in a study then the Gompertz curve resulted in best fits. The Gompertz curve as best suited curve for domestic birds has also been suggested from a mechanistic standpoint [82, 83]. Partial agreements were found in the present study, where for the pooled fit, the Richards followed by the Gompertz curve performed best in laying hens. As the laying hen data used in this study were mainly from industrial standards, it may be assumed that nutrition was not a limiting factor. For broiler chickens the Richards and West curves performed best; for the breed-specific fits, the Rich_d_ and Gomp_breed_ curves performed similarly well in laying hens, but not better than the Rich_breed_ fit, whereas in broilers, both the Rich_d_ and Rich_breed_ fits showed clear improvements over the other curve models.

The Konya Merino sheep publication [61] used in this study deemed a polynomial or Gompertz model best-fitting. Other examples of Gompertz fits agreeing with sheep data can be found in studies of six European sheep breeds reared under non-limiting conditions [84, 85]. In a meta study of the BP model in goat and sheep [86], the Monomolecular curve was deemed to fit best to various sheep breed data, among the named models. BP curves outside the named models fitted the individual sheep breeds data better, however. Compare to the present study where the Monomolecular curve achieved best fit for the pooled model in sheep, but did not converge for a number of individual breeds.

Overall, in literature, curves with flexible inflection point such as the Richards curve achieved better fits compared to less flexible curve models when fitting to breed-specific data [81, 86], with exceptions [80]. This was also reflected in the results of the breed-specific fits of the present study, where the Richards curve outperformed other named models even when fitting simultaneously to multiple breeds.

### Limitations of the study

Individuum-level growth data were sparsely available in literature, thus no such data were used in this study, consequently, the random effects involving the repeated measurements from individual animals were not accounted for. For beef cattle and sheep, available breed data was not balanced with regard to sex of animals. Presence of weight differences due to sex dimorphism is well established for those species [87, 88]. While for the breed-specific fits, sex of each group of breed was stated and fitted separately, in the pooled fit, the use of mixed sex data may lead to bias.

Heteroscedasticity was removed in most species in the pooled model, though it remained in sheep. This was due to sheep breed data covering time spans of variable lengths, thus introducing bias caused by different breeds. New continuous measurements throughout growth periods could fill these data gaps. Non-normality of the residuals was detected in a few species, which may make computation of confidence and prediction intervals invalid; however, for application in PBK models, these are not required.

### Richards family of curves

The Richards and Monomolecular curves tended to over- or underpredict important physiological characteristics such as adult and birth weight. While the Richards curve was more prone to overfitting to data due to additional flexibility, the Monomolecular curve was unable to take into account the initial delay in growth due to a lack of an inflection point.

The Von Bertalanffy curve posited growth rate as a difference between anabolic and catabolic processes [89], with the anabolic rate proportional to BW^2/3^, while the West curve assumed growth rate to be the difference between anabolic processes and maintenance of existing tissue, with the anabolic rate proportional to BW^3/4^. These two values of the exponent *d* were based either on the assumption that anabolic rate followed allometric observations of a BW-to-body surface rule (*d = 2/3*) [90] or of the oxygen consumption rate (*d = 3/4*) [91]. Due to its relatively recent development as growth curve in 2001, the West curve has not been widely used yet compared to the other named models, and it has been criticized for not differing meaningfully from the Von Bertalanffy curve [92, 93].

## Conclusions

Growth was indicated to have a strong impact on the organ concentrations, and was shown to be an added value of PBK models [7, 8]. The obtained curves in this paper allow the study of long-term exposure using a generic PBK model while accounting for growth in livestock species. The extensive comparison of curves facilitates risk assessment by allowing a statistically informed model choice. Ultimately, whether a curve model is chosen due to mechanistic or statistical considerations is up to the modeler. The presented approach and database can be extended to cover more species.

It has been shown in the pooled fits that named models with fixed inflection points can return similar statistical results as the flexible Richards curve. Thus, it is possible to use a more parsimonious growth model instead of the Richards curve to achieve desirable fits. The West and Von Bertalanffy curves consistently performed better than the Monomolecular or Gompertz curves in cattle and broilers, whereas in laying hens, the Gompertz curve appeared to be a good fit. A special case were the sheep data where the Monomolecular curve returned a better fit. For beef and dairy cattle, it was found that there were no differences in curve shape of the Richards curve. While there were significant differences between the pooled and breed-specific fits, it yet needs to be studied whether those differences make a significant impact when integrated into PBK models.

## Supplementary Information

Supplementary Material 1

Supplementary Material 2

## Data Availability

The datasets generated and/or analysed during the current study are available in the Zenodo repository, at https://doi.org/10.5281/zenodo.13886573

## Abbreviations

AF: Additional file
AIC: Akaike information criterion
BIC: Bayesian information criterion
BP: Bertalanffy-Püttner
BW: Body weight
ELS: Extended least squares
HF: Holstein Friesian
LR: Likelihood-ratio
PBK model: Physiologically based kinetic model
RGR: Relative growth rate
RMSE: Root mean squared error
WLS: Weighted least squares
