# Supplementary material for: A general Richards family growth curve fit in European livestock for use in physiologically-based toxicokinetic modelling
Source: BMC Vet Res. 2025 Dec 4;22:16. doi: 10.1186/s12917-025-04982-8 (PMC12794480; doi:10.1186/s12917-025-04982-8)
Supplement: Supplementary file 1 — Supplementary Material 1 [file 12917_2025_4982_MOESM1_ESM.docx]

A general Richards family growth curve fit in European livestock for use in physiologically-based toxicokinetic modelling

D Inauen^1*^, LS Lautz^2^, AJ Hendriks^3^, R Gehring^1^

^1^ Utrecht University, Faculty for Veterinary Medicine, Institute for Risk Assessment Sciences, One Health Pharmacology, Yalelaan 104-106, 3584 CM Utrecht, the Netherlands

^2^ ESQlabs GmbH, Am Sportplatz 7, Saterland 26683, Germany

^3^ Radboud University Nijmegen, Department of Environmental Science, Heyendaalseweg 135, 6525 AJ Nijmegen, the Netherlands

Additional file 1

# Model specification

The following was adapted from [1], [2].

## Pooled model

Let f(t, A, W0, kU, d) be the Richards curve dependent on time and the four curve parameters. Let β_A_, β_W0_, β_kU_, β_d_ be fixed effects to be estimated. Let i =1…M with M the number of groups (breeds) and j = 1…n_i_ with n_i_ the number of observations in group i. Then y_ij,_ the j-th observation of the i-th group, is given as

$$y_{\mathrm{ij}}= f\left( t_{\mathrm{ij}}, A_{i},W_{0i},\mathrm{kU}_{i}, d_{i} \right)+\varepsilon_{\mathrm{ij}}$$

$$A_{i}=\beta_{A}, W_{0i}=\beta_{W0}, \mathrm{kU}_{i}=\beta_{\mathrm{kU}},d_{i}= \beta_{d}$$

with t_ij_ the time point of the observation and $\epsilon_{ij}$ a residual term. Note that the curve parameters do not vary with i. For **ε**_i_. the vector of residuals of group i, then **ε_i_** ~ N(0, σ^2^**Λ**_i_) with σ^2^**Λ**_i_ a variance-covariance matrix decomposable into a variance and correlation structure: σ^2^**Λ**_i_ = σ^2^**G**_i_**C**_i_**G**_i_. σ is the residual standard error, **G**_i_ is a diagonal matrix with positive diagonal values where (**G**_i_)_jj_ = g(x_ij_,θ) = |x_ij_|^θ^, specifying the variance structure. x_ij_ denotes the covariate used in the weighting function, in this case the fitted values $\hat{W}\left( t \right)$ of the unweighted regression. **C**_i_ is a correlation matrix with (**C**_i_)_jj’_ = φ^|tij- tij’|^ for j, j’ = 1…n_i_ and t_ij_ the time points. For other curves, the implementation is the same, but without the d parameter.

## Rich_d_ model

The breed-specific models were implemented without an intercept, that is, no reference group was chosen; code syntax: gnls(…, params = list(A ~ Breed -1,…)). Let **β**_A_, **β**_W0_, **β**_kU_ be vectors of length M of fixed effects and β_d_ a single fixed effect, to be estimated. Let **v**_i_ = (v_i,m_)_m_ be indicator vectors such that v_i,m_ =1 for m=i, and 0 otherwise. Then y_ij,_ the j-th observation of the i-th group, is given as

$$y_{ij}= f\left( t_{ij}, A_{i},{W_{0}}_{i},kU_{i}, d_{i}, \mathbf{v}_{i} \right)+\varepsilon_{ij}$$

$$A_{i}=\sum_{m=1}^{M} \beta_{A,m}*v_{i,m}$$

$$W_{0i}=\sum_{m=1}^{M} \beta_{W0,m}*v_{i,m}$$

$$kU_{i}=\sum_{m=1}^{M} \beta_{kU,m}*v_{i,m}$$

$$d_{i}=\beta_{d}$$

Note that d_i_ does not vary by group, thus is constant and shared between all groups (breeds). **ε**_i_ is the vector of residuals of group i, **ε_i_** ~ N(0, σ^2^**I**_i_) with **I**_i_ the n_i_ × n_i_ identity matrix.

## Rich_breed_ model

Like the Rich_d_ model, with the difference that **β**_d_ is a fixed effects vector to be estimated, and

$$d_{i}=\sum_{m=1}^{M} \beta_{d,m}*v_{i,m}$$

This model is equivalent to fitting a curve to each breed separately. For other curves, the implementation is the same, but without the d parameter.

# Evaluation metrics

The Akaike information criterion (AIC) and Bayesian information criterion (BIC) were defined as

$$AIC = -2LL +2k$$

$$BIC = -2LL +log(n)k$$

respectively, with LL the maximized log-likelihood of the model, k the number of parameters of the model and n the number of observed data points.

The root mean squared error (RMSE) was defined as

$$\mathrm{RMSE}=\sqrt{\sum_{i=1}^{n} \frac{\left( y_{i}-\hat{y}_{i} \right)^{2}}{\left( y_{i}-\bar{y} \right)^{2}}}$$

with $\hat{y}_{i}$ the fitted value and $\bar{y}$ the observed mean.

# Diagnostic plots

## Beef cattle


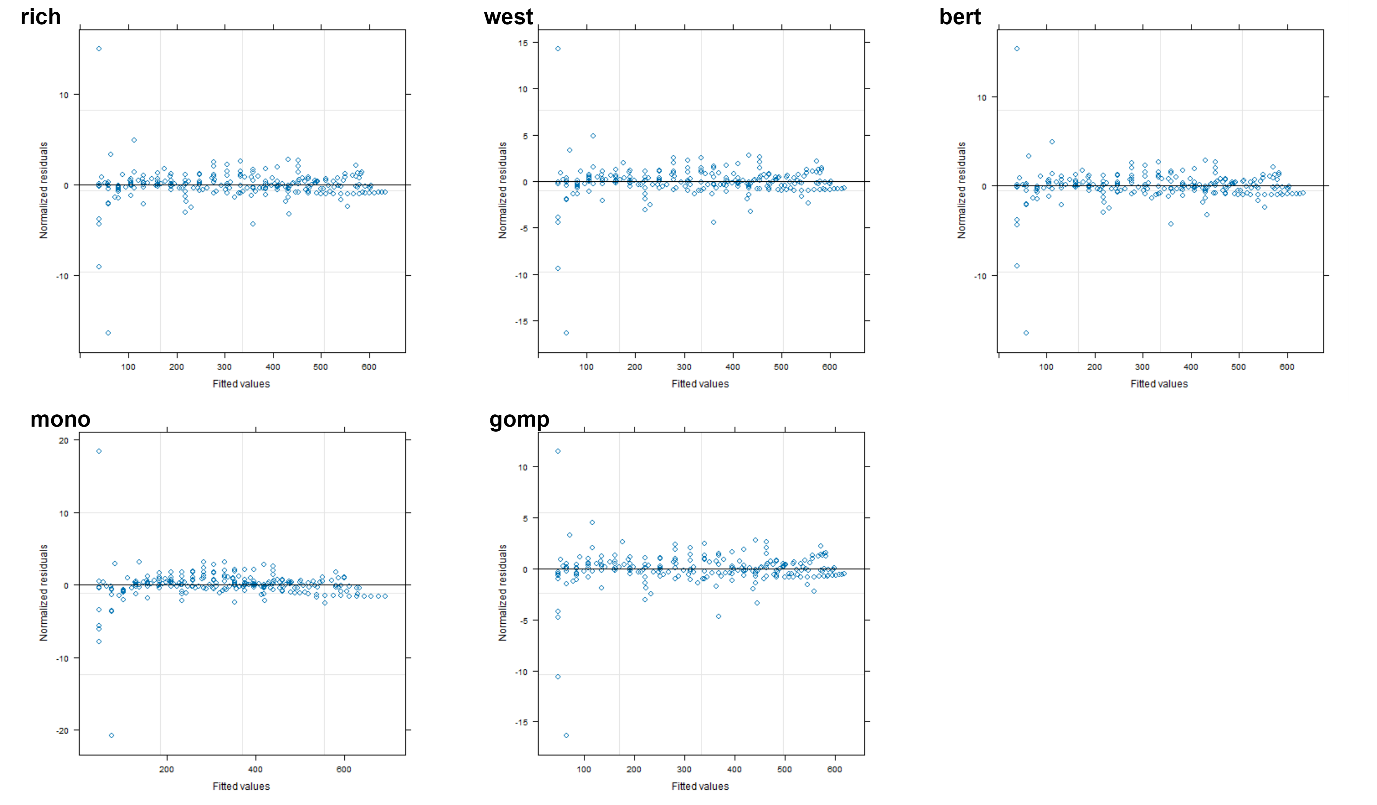


Figure 1 Fitted versus normalized residuals best fits pooled model beef cattle


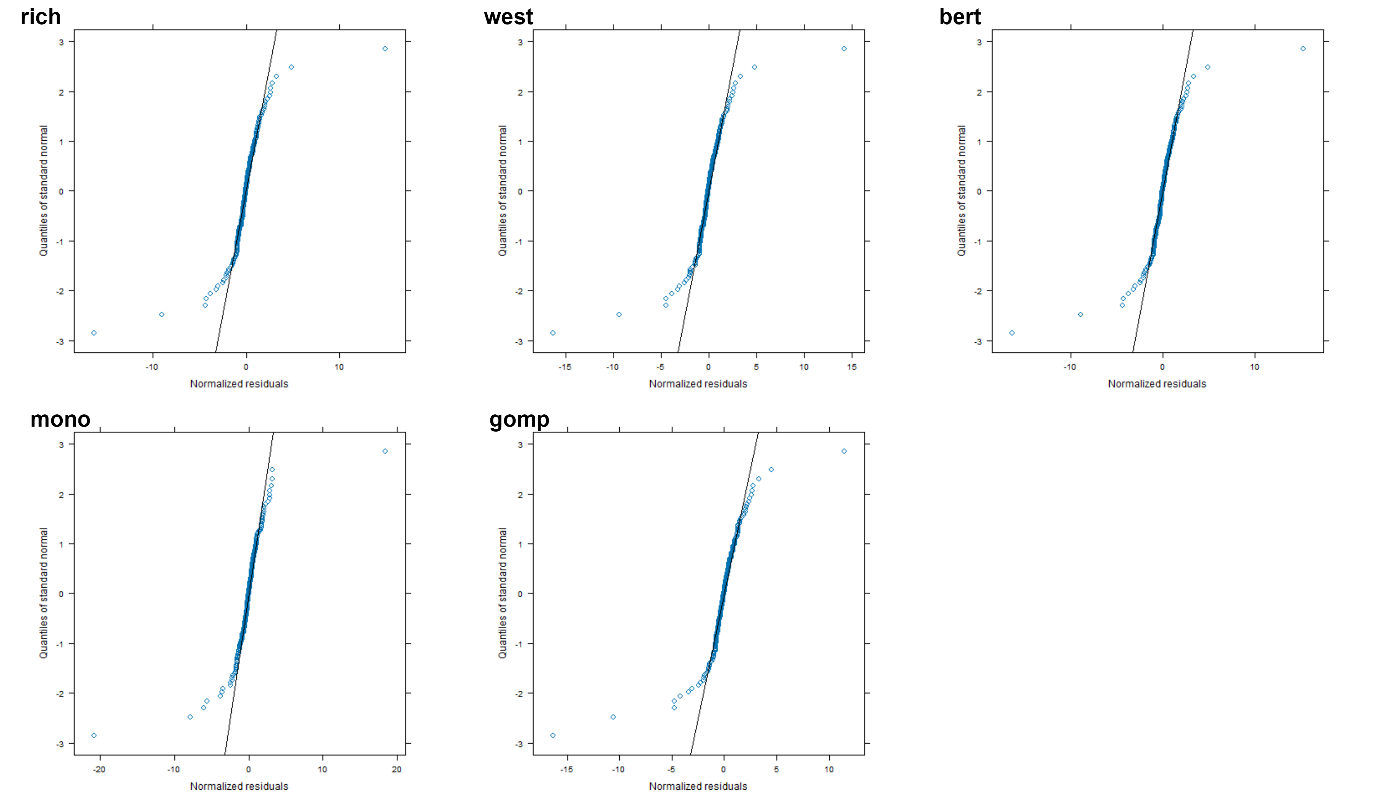


Figure 2 QQ plots normalized residuals best fits pooled model beef cattle


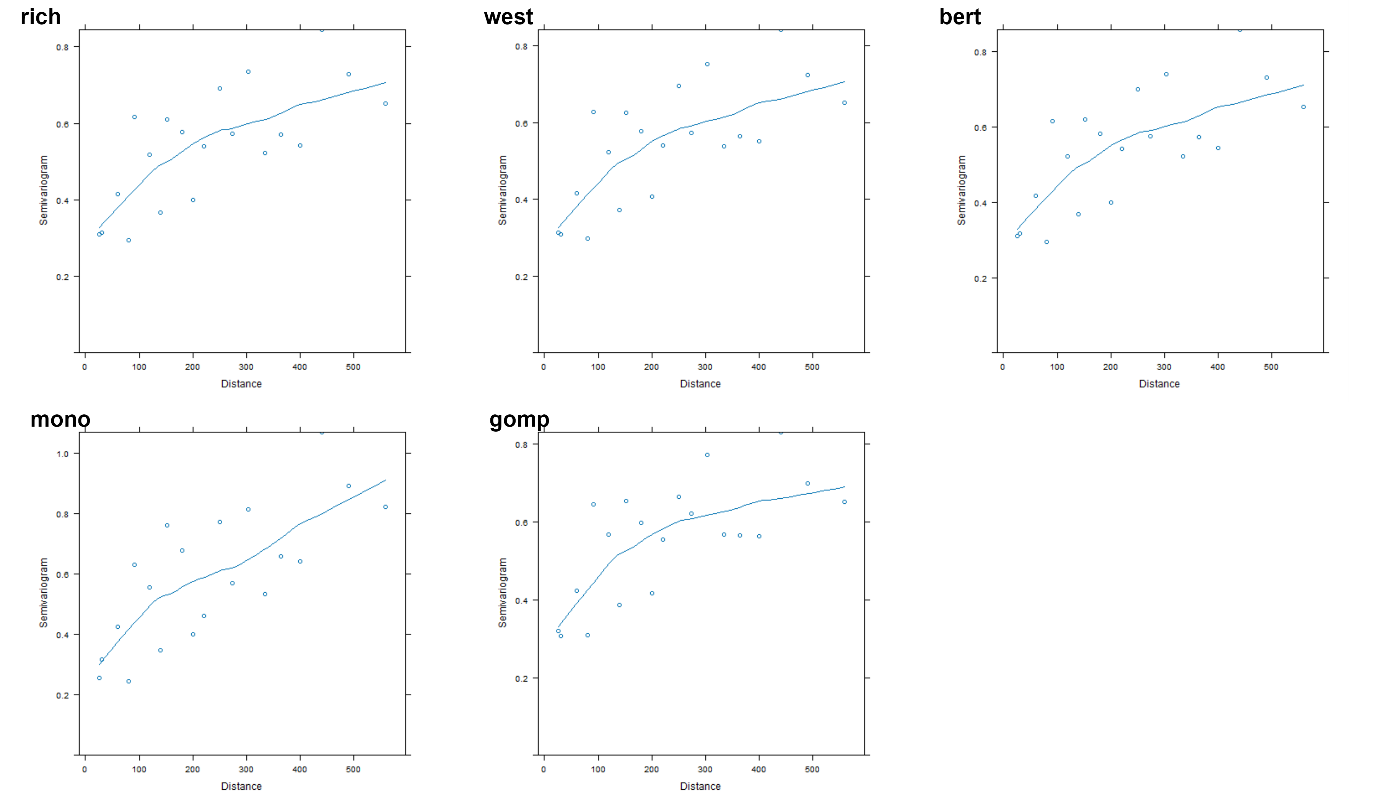


Figure 3 Temporal semivariograms best fits pooled model beef cattle

Dairy cattle
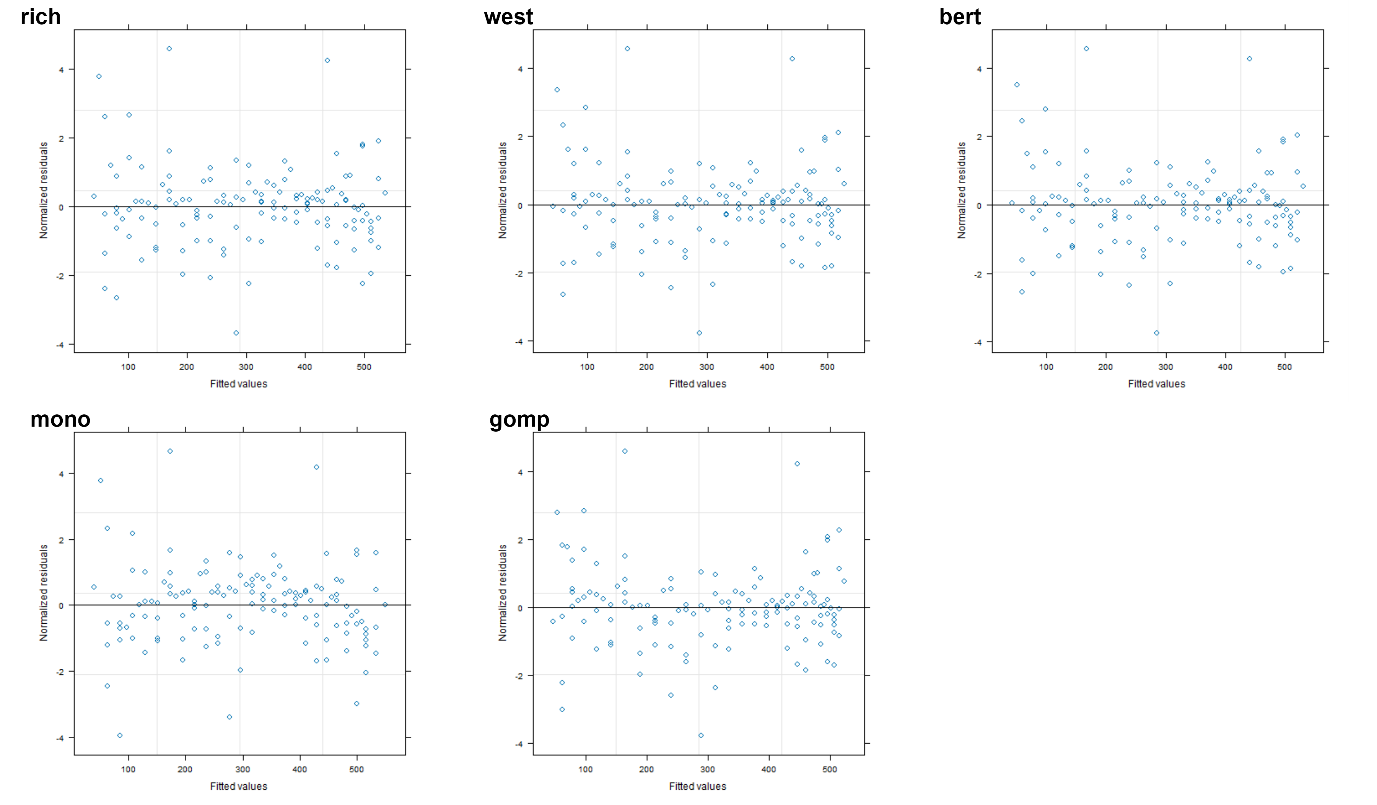


Figure 4 Fitted versus normalized residuals best fits pooled model dairy cattle


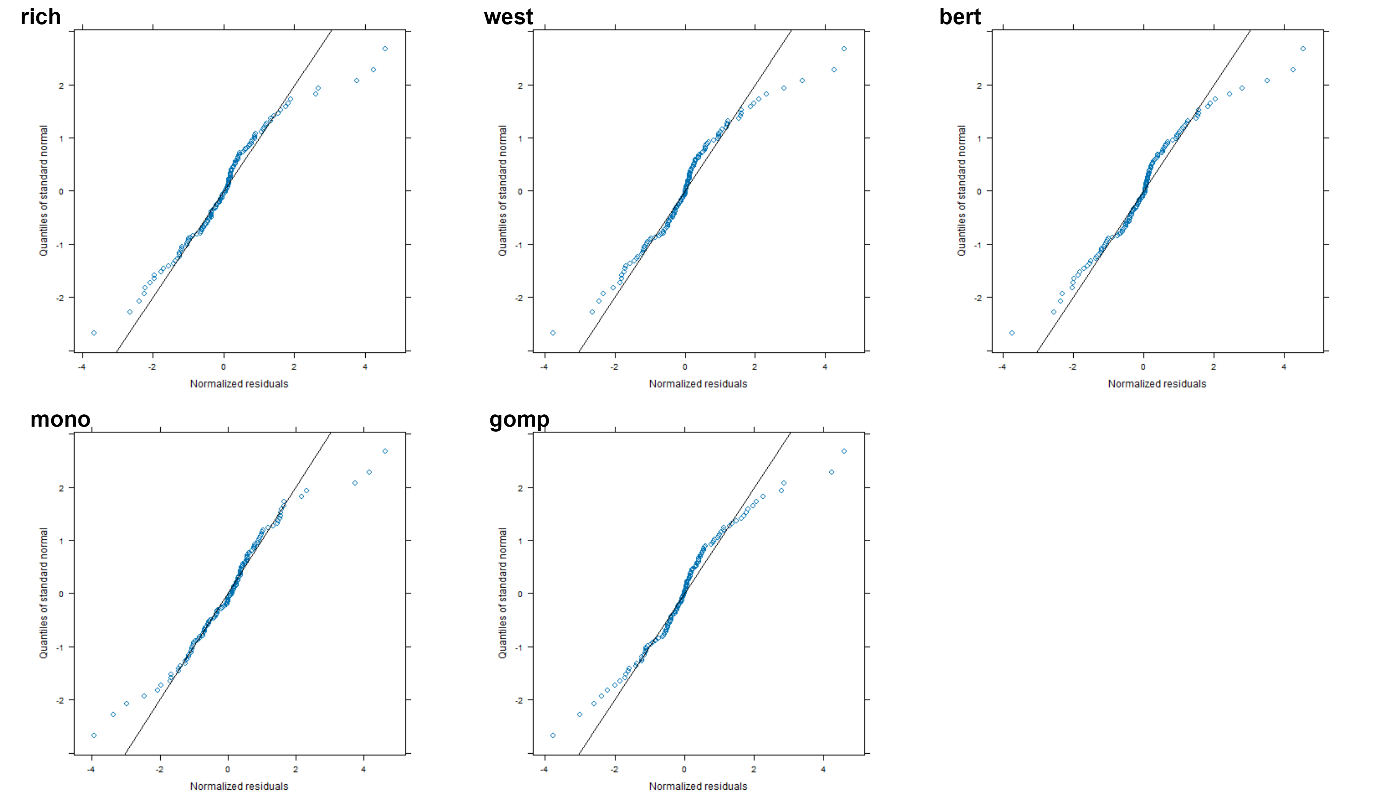


Figure 5 QQ plots normalized residuals best fits pooled model dairy cattle


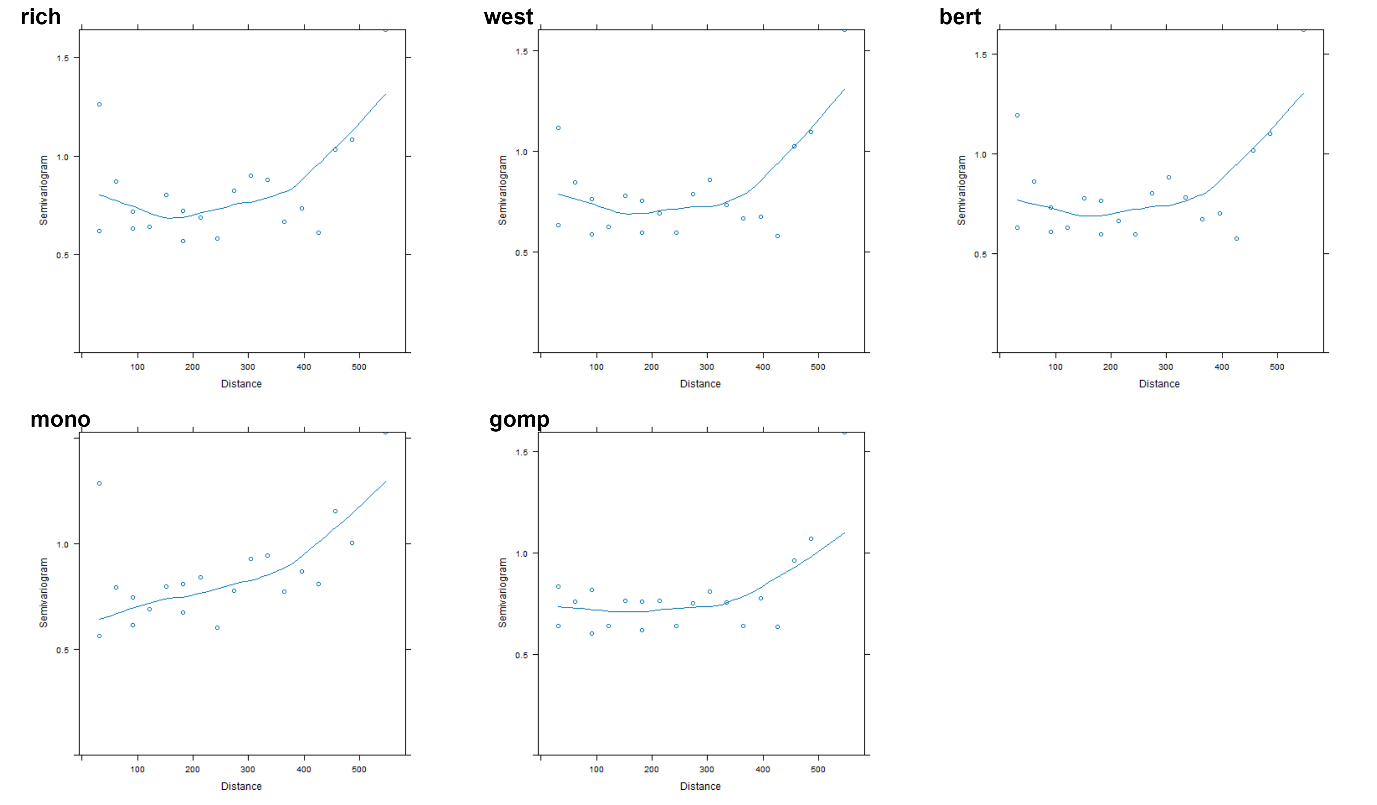


Figure 6 Temporal semivariograms best fits pooled model dairy cattle

## Laying hens


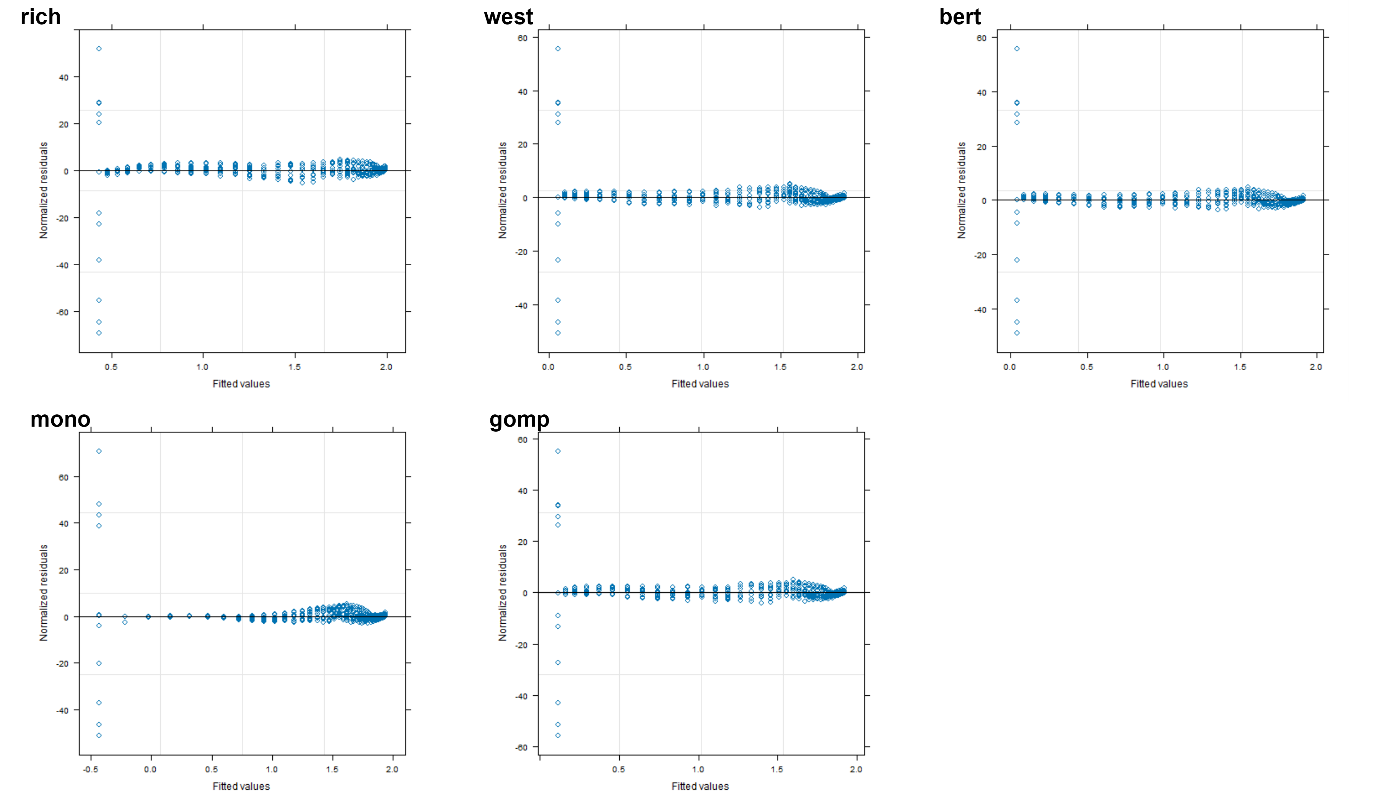


Figure 7 Fitted versus normalized residuals pooled model laying hen


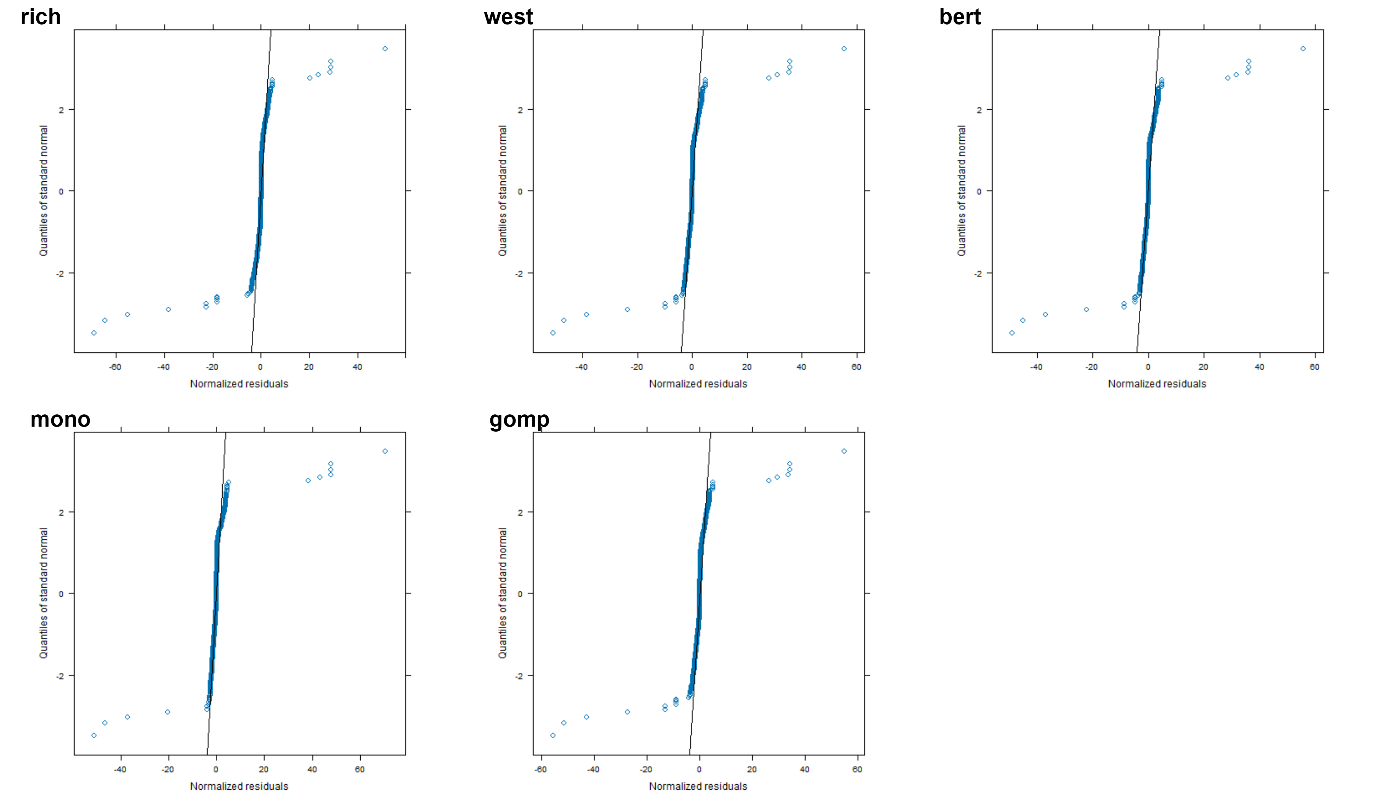


Figure 8 QQ plots normalized residuals best fits pooled model laying hen


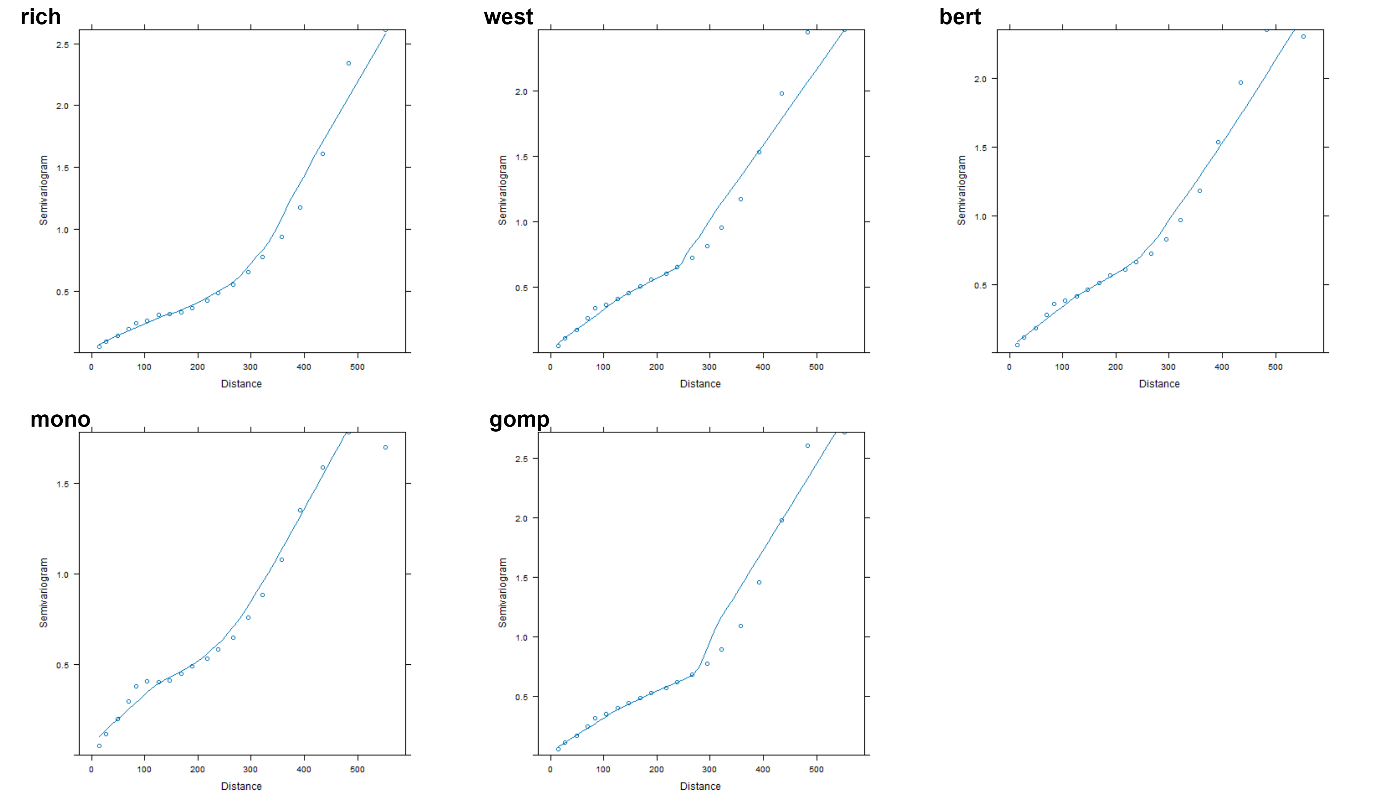


Figure 9 Temporal semivariograms best fits pooled model laying hen

## Male broiler chicken


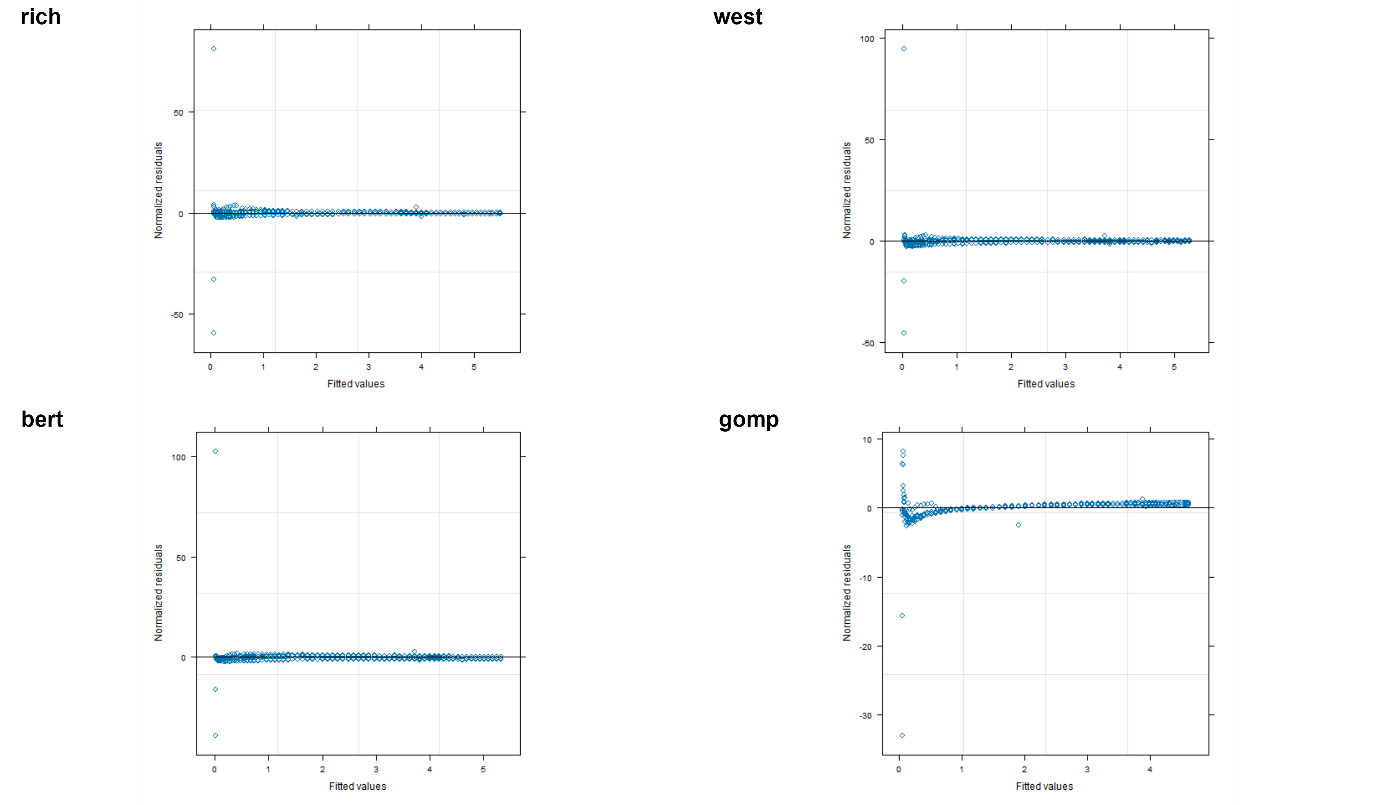


Figure 10 Fitted versus normalized residuals pooled model broiler chicken M


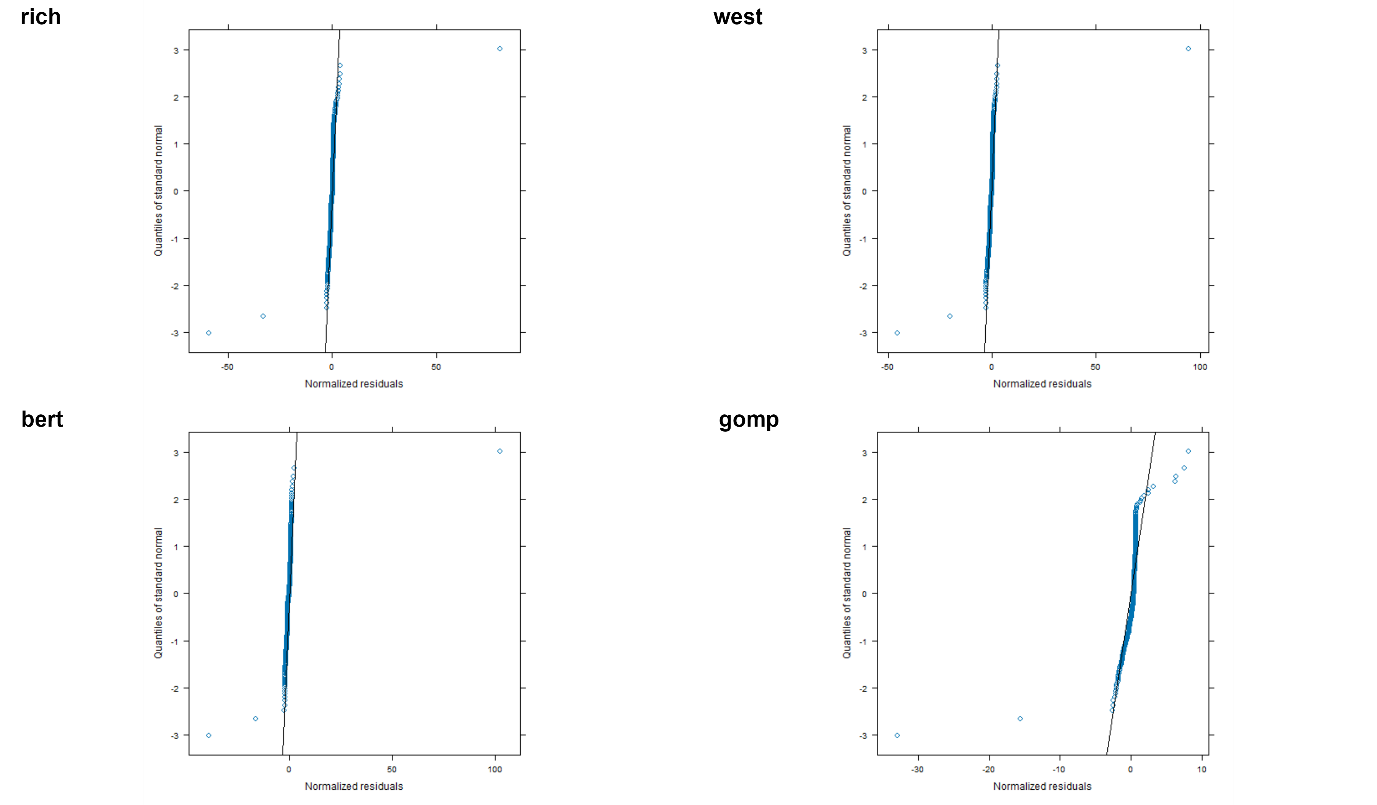


Figure 11 QQ plots normalized residuals pooled model broiler chicken M


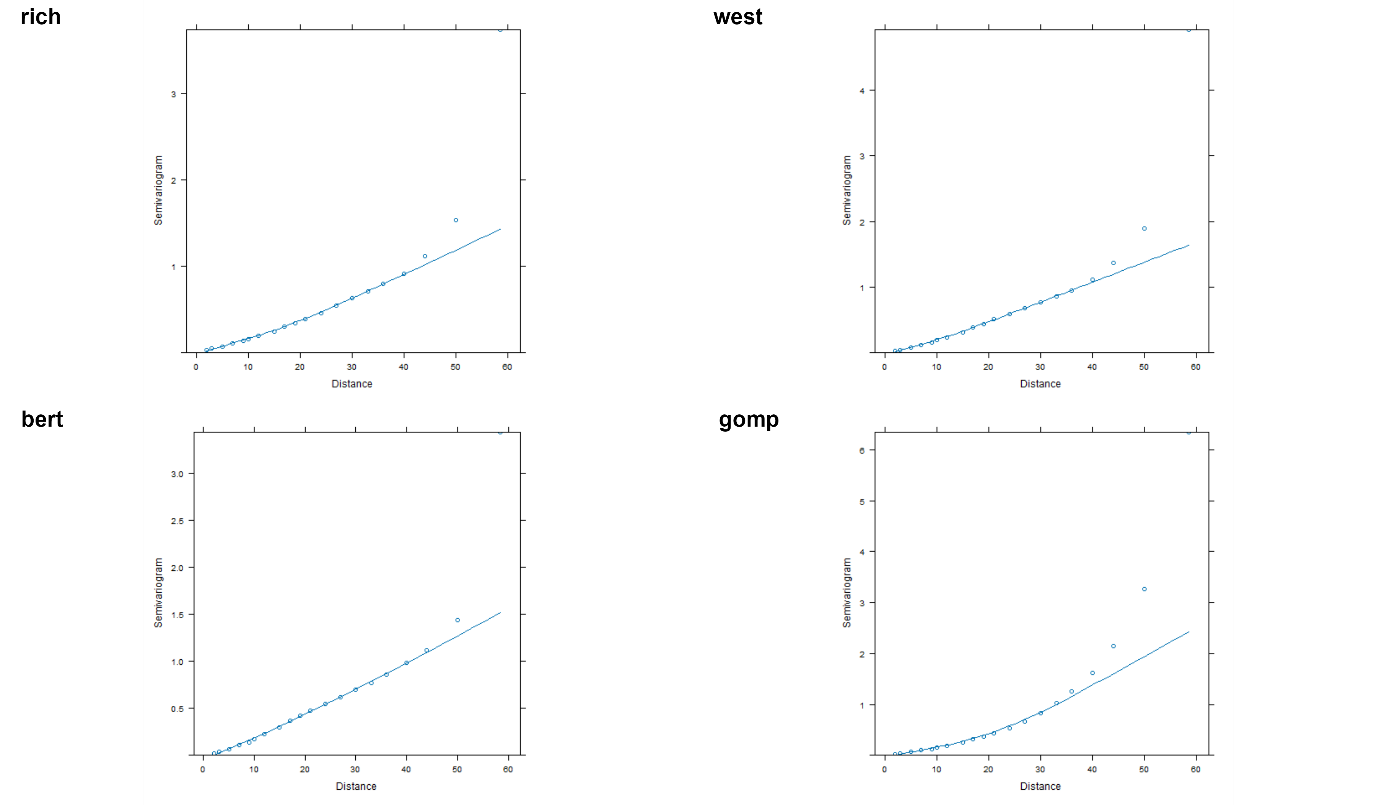


Figure 12 Temporal semivariograms best fits pooled model broiler chicken M

## Female broiler chicken


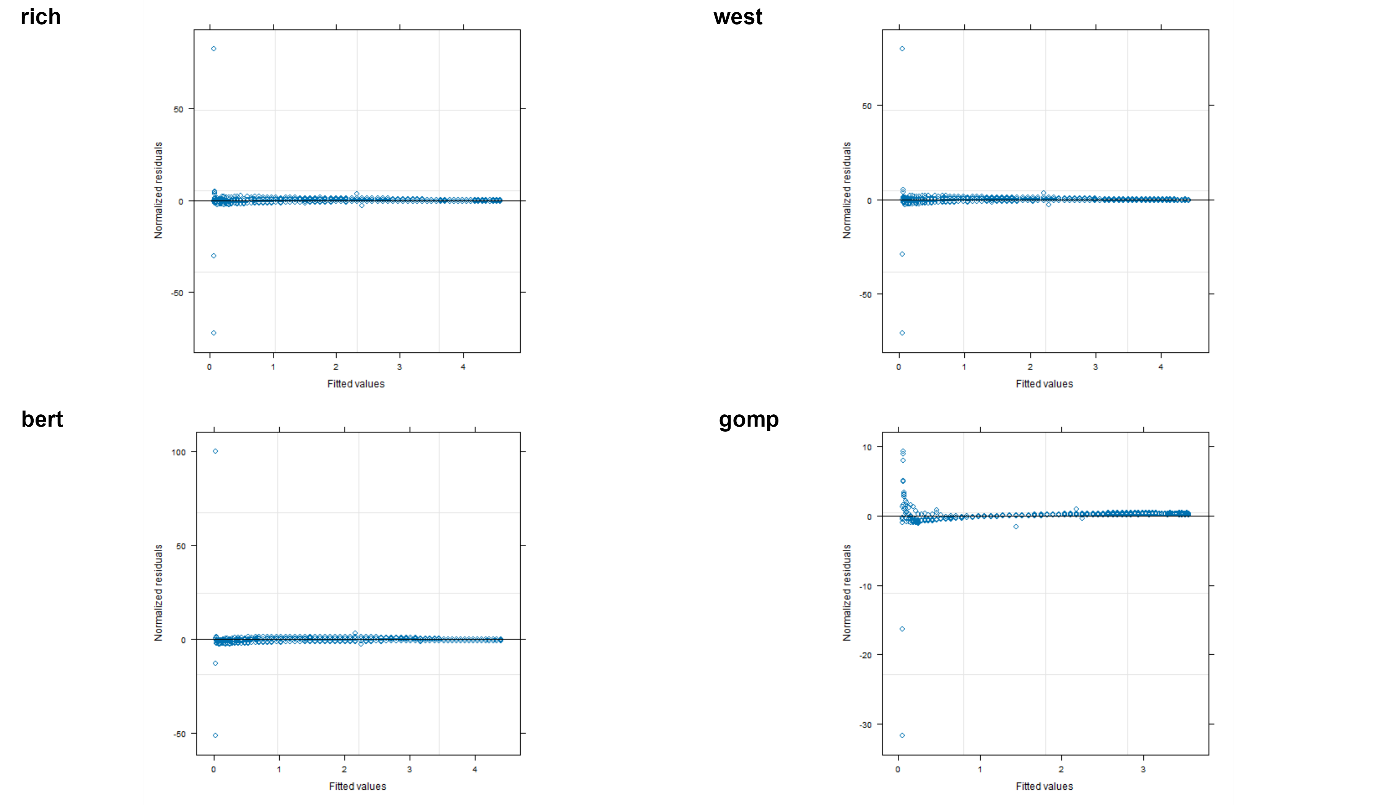


Figure 13 Fitted versus normalized residuals pooled model broiler chicken F


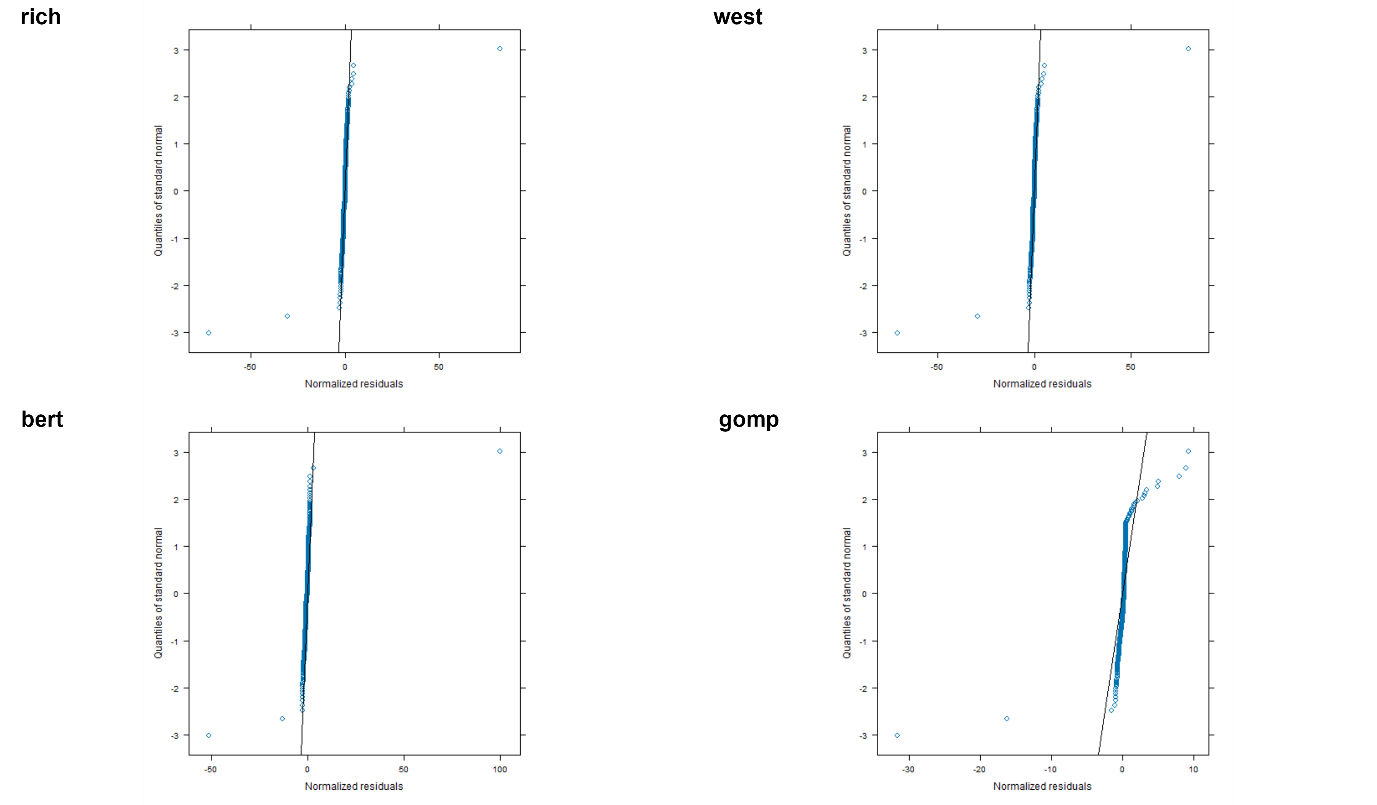


Figure 14 QQ plots normalized residuals pooled model broiler chicken F


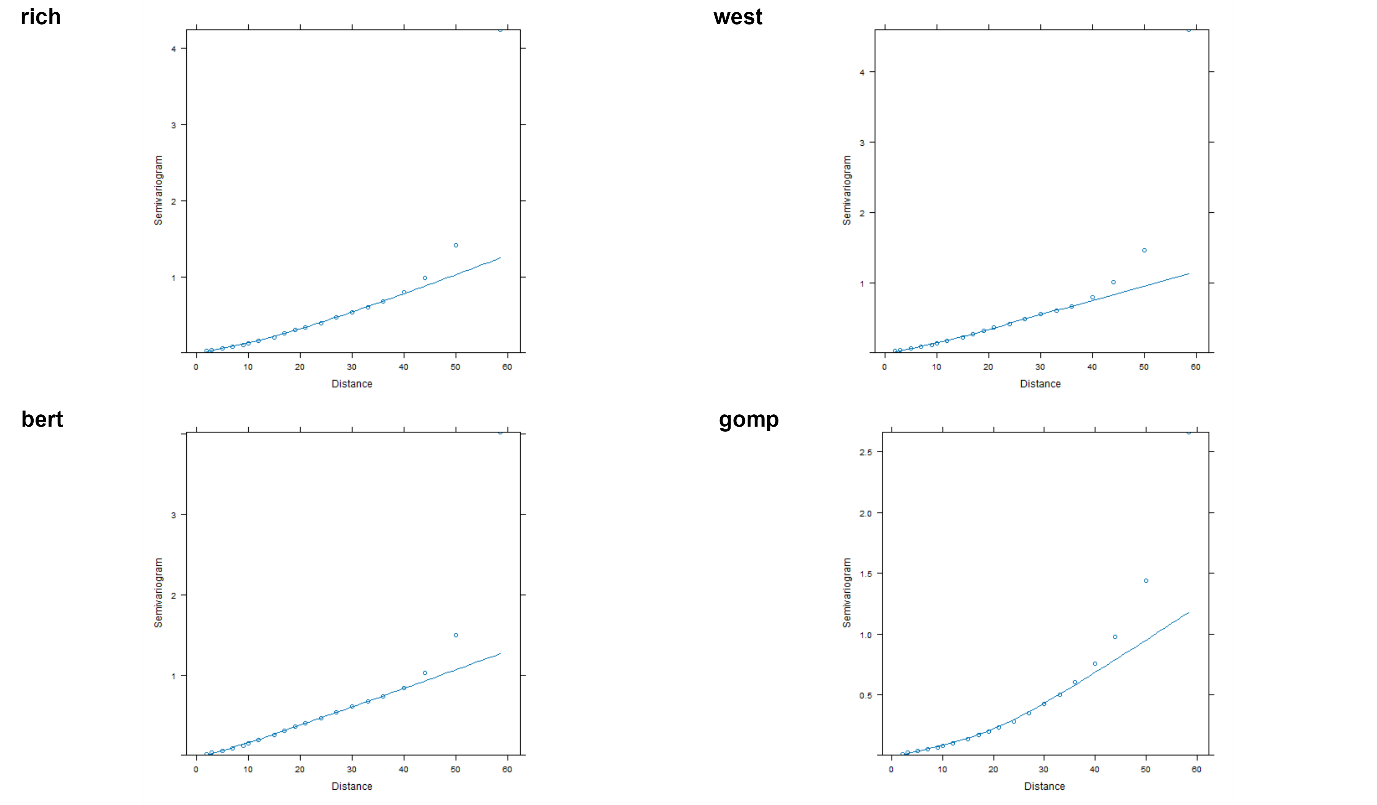


Figure 15 Temporal semivariograms best fits pooled model broiler chicken F

## Sheep


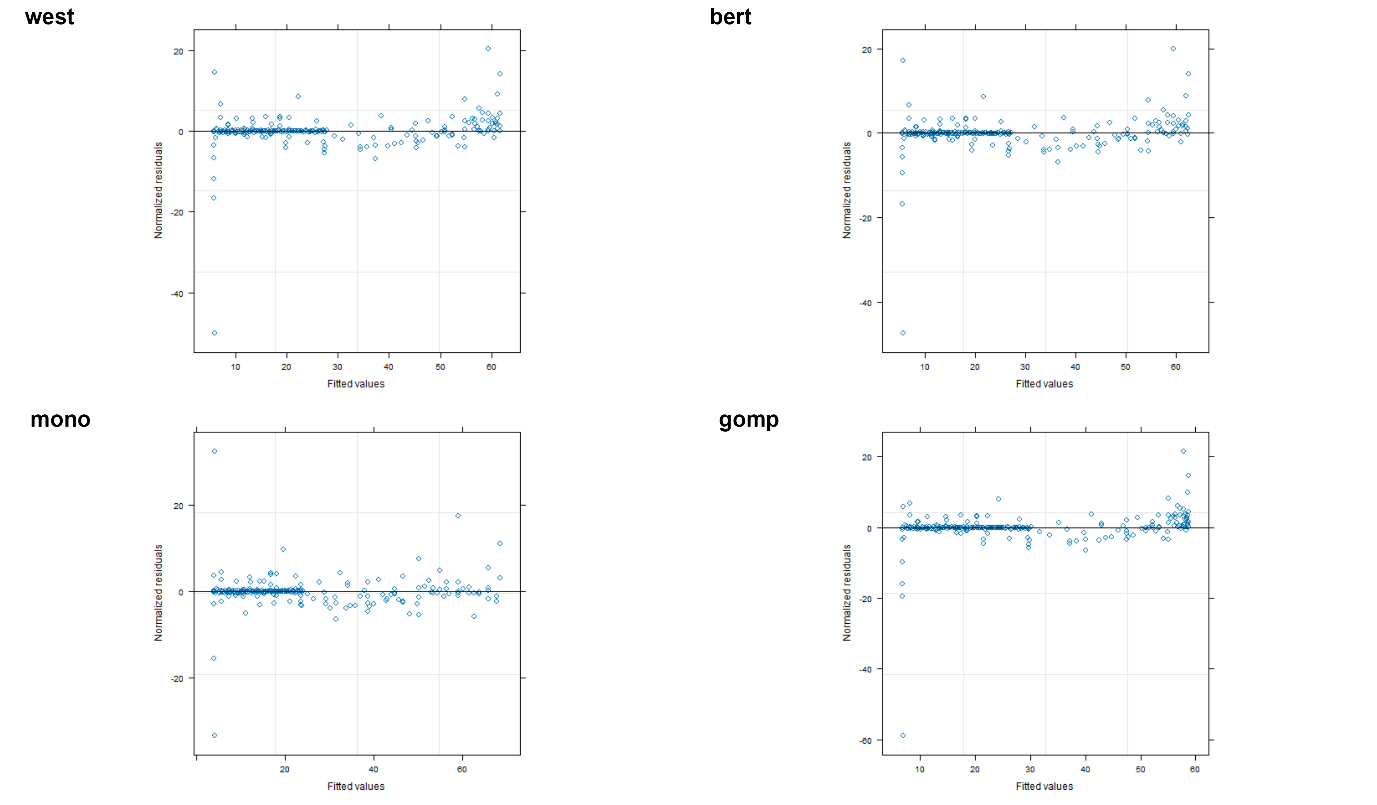


Figure 16 Fitted versus normalized residuals pooled model sheep


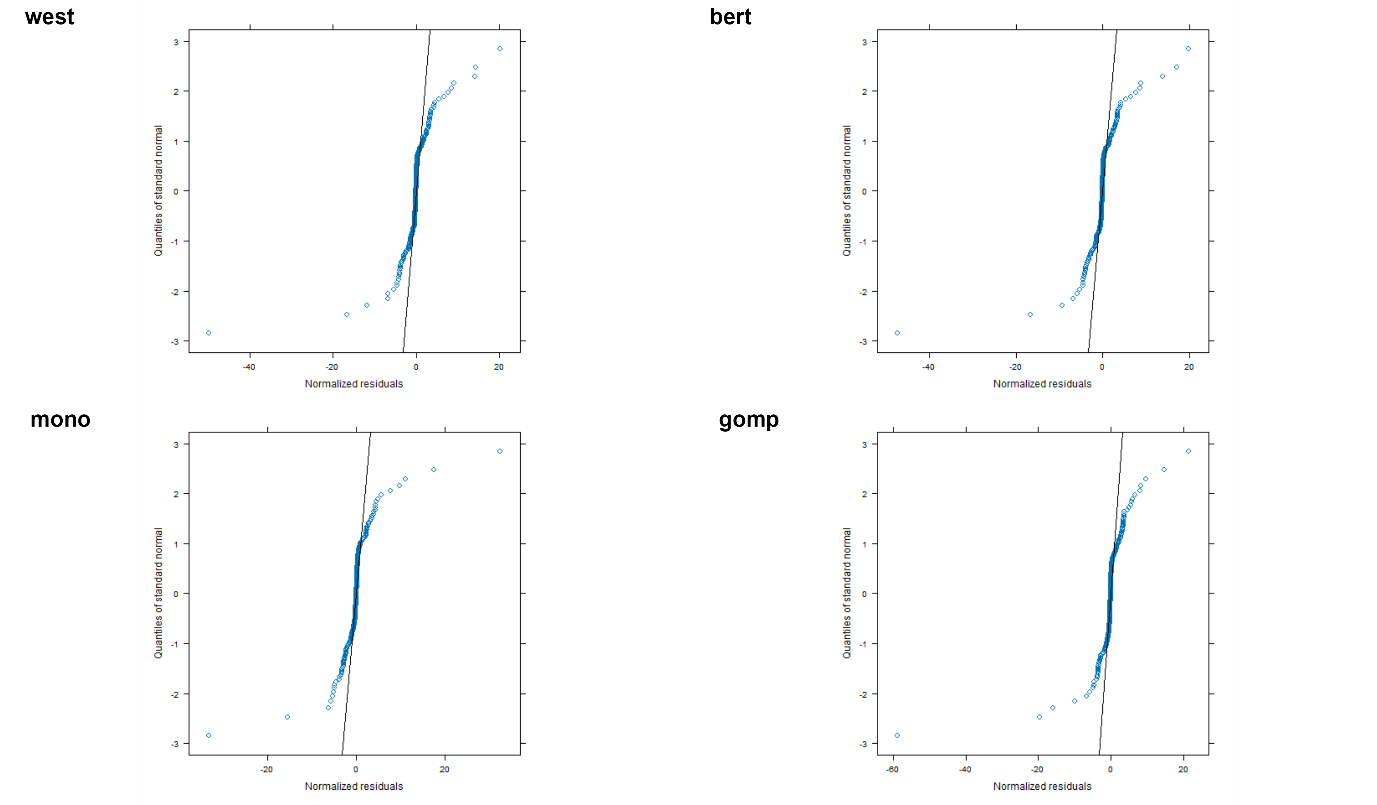


Figure 17 QQ plots normalized residuals pooled model sheep


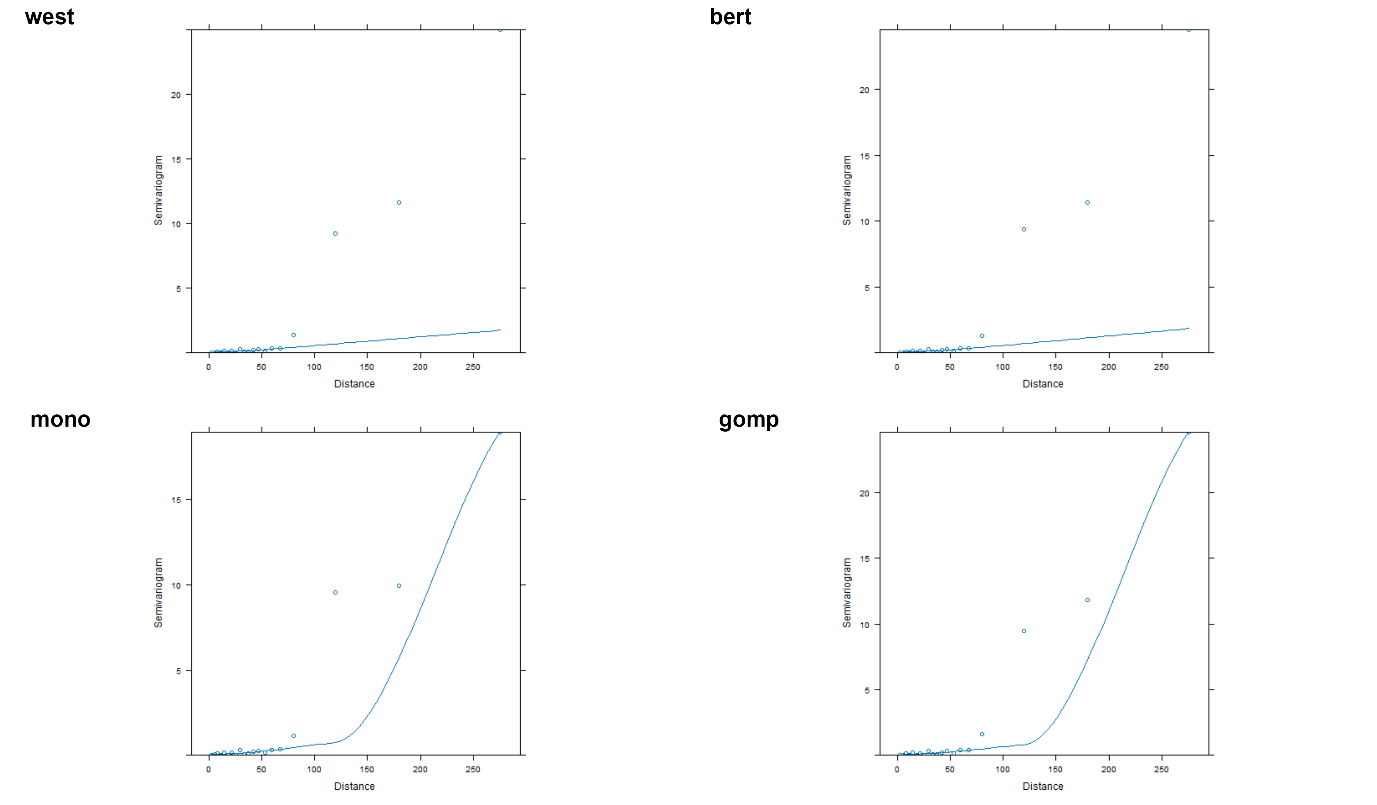


Figure 18 Temporal semivariograms best fits pooled model sheep

# Breed-specific fits BIC and RMSE

Table 1 BICs of curve fits when varying parameters by breed.

| Species | Rich_d_ | d (SE) | West_breed_  (d=3/4) | Bert_breed_  (d=2/3) | Mono_breed_  (d=0) | Gomp_breed_  (d↘1) | Rich_breed_ | d (Range) |
| --- | --- | --- | --- | --- | --- | --- | --- | --- |
| Beef cattle | 1991.5 | 0.38±0.09 | 2001.5^‡^ | 1995.8^‡^ | 2268.5^‡^ | 2022.8^‡^ | 2026.7 | 0.09-0.56 |
| Dairy cattle | 1042.0 | 0.58±0.12 | 1039.2 | 1037.6 | 1066.7^‡^ | 1048.6^‡^ | 1059.1 | 0.22-1.15 |
| Laying hen | -9103.8 | 0.99±0.01 | -8842.0^‡^ | -8620.2^‡^ | -5280.5^‡^ | -9110.5 | -9080.5* | 0.76-1.40 |
| Broilers M | -3096.6 | 0.84±0^+^ | -2441.2^‡^ | -1977.7^‡^ | NA | -2131.5^‡^ | -3222.4* | 0.77-0.87 |
| Broilers F | -3248.4 | 0.82±0^+^ | -2846.8^‡^ | -2253.4^‡^ | NA | -2162.9^‡^ | -3563.8* | 0.79-0.84 |
| Sheep | NA | NA | 1025.9 | 1018.0 | NA | 1048.4 | NA | NA |

d: Richards curve shape parameter. Estimates of d are added for the Rich_d_ and Rich_breed_ models ^‡^Fit significantly worse than Rich_d_. *Fit significantly better than Rich_d_. Instances where no convergence was achieved are indicated by “NA”. 0^+^ indicates positive values smaller than 0.01. Dairy cattle and laying hen were without Jersey and White Leghorn breeds, respectively.

Table 2 RMSEs of curve fits when varying parameters by breed.

| Species | Rich_d_ | d (SE) | West_breed_  (d=3/4) | Bert_breed_  (d=2/3) | Mono_breed_  (d=0) | Gomp_breed_  (d↘1) | Rich_breed_ | d (Range) |
| --- | --- | --- | --- | --- | --- | --- | --- | --- |
| Beef cattle | 12.4 | 0.38±0.09 | 12.8^‡^ | 12.6^‡^ | 22.8^‡^ | 13.4^‡^ | 12.0 | 0.09-0.56 |
| Dairy cattle | 8.4 | 0.58±0.12 | 8.5 | 8.5 | 9.4^‡^ | 8.8^‡^ | 8.2 | 0.22-1.15 |
| Laying hen | 0.02 | 0.99±0.01 | 0.02^‡^ | 0.02^‡^ | 0.06^+‡^ | 0.02^+^ | 0.02* | 0.76-1.40 |
| Broilers M | 0^+^ | 0.84±0^+^ | 0.01^‡^ | 0.02^‡^ | NA | 0.01^‡^ | 0^+^* | 0.77-0.87 |
| Broilers F | 0^+^ | 0.82±0^+^ | 0.01^‡^ | 0.01^‡^ | NA | 0.01^‡^ | 0^+^* | 0.79-0.84 |
| Sheep | NA | NA | 1.7 | 1.7 | NA | 1.8 | NA | NA |

d: Richards curve shape parameter. Estimates of d are added for the Rich_d_ and Rich_breed_ models ^‡^Fit significantly worse than Rich_d_. *Fit significantly better than Rich_d_. Instances where no convergence was achieved are indicated by “NA”. 0^+^ indicates positive values smaller than 0.01. Dairy cattle and laying hen were without Jersey and White Leghorn breeds, respectively.

# Jersey dairy cattle fits


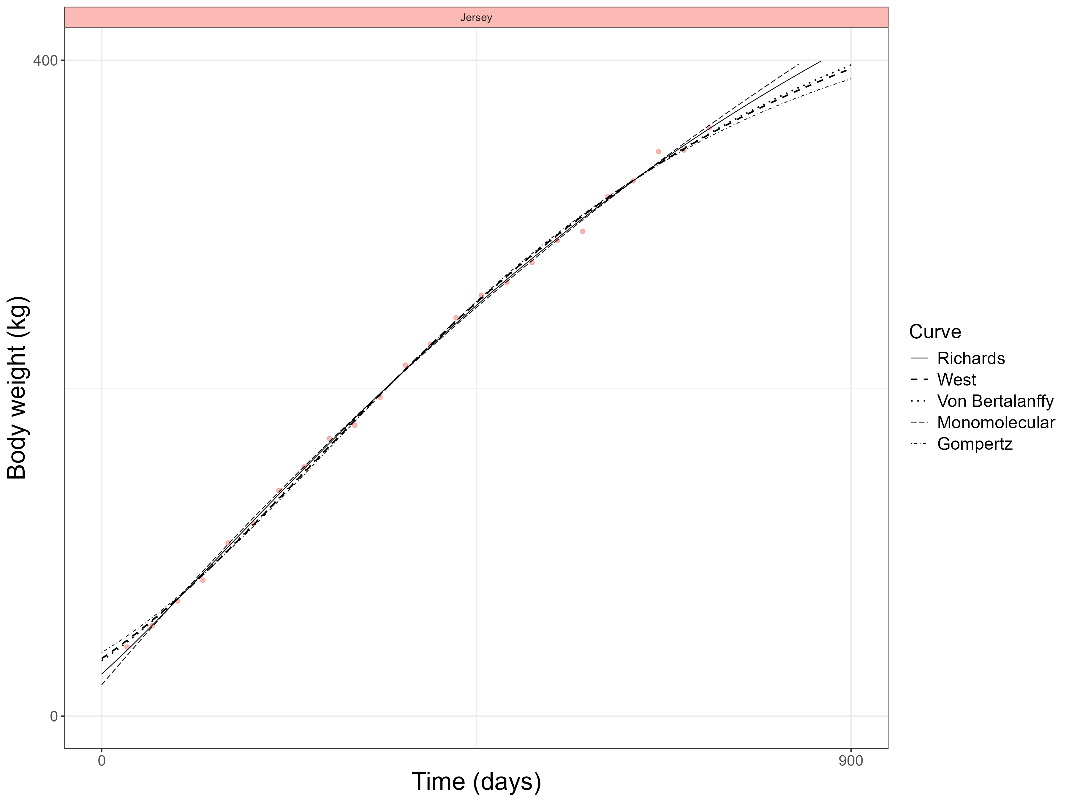


Figure 19 Breed-specific growth curve fits in Jersey dairy cattle of the Richards (solid), West (dashed), Von Bertalanffy (dotted), Monomolecular (longdash), and Gompertz (dotdash) curves.

# White Leghorn laying hen fits


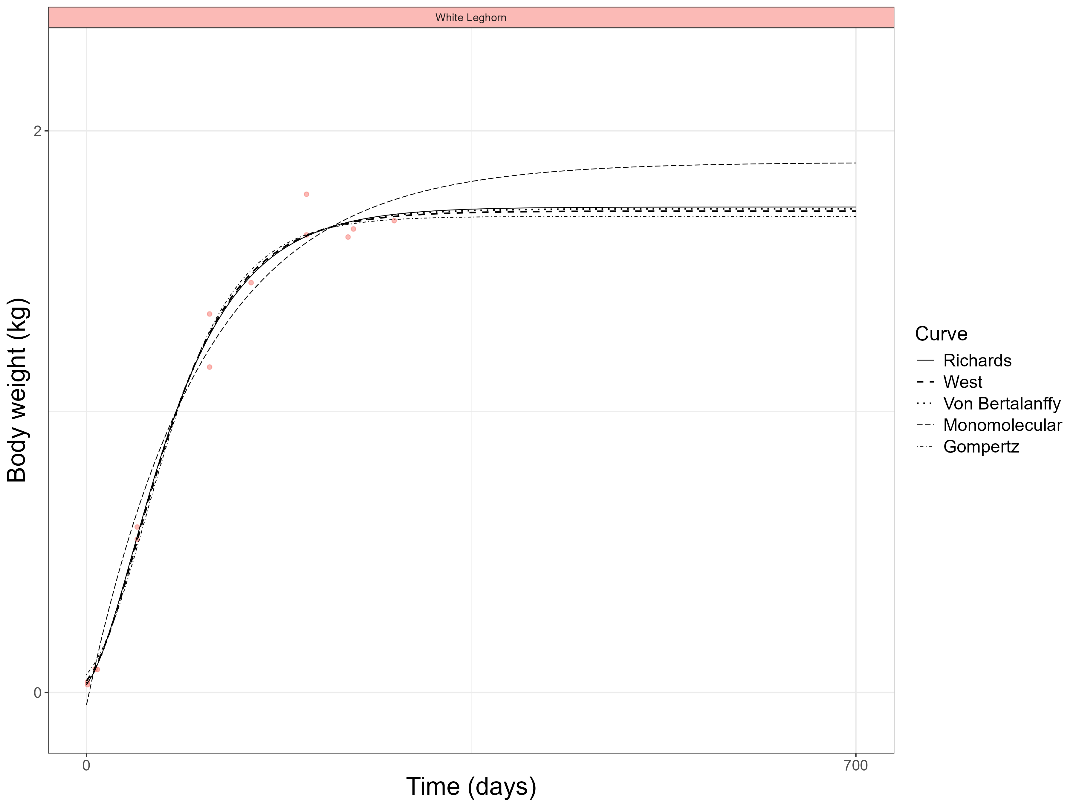


Figure 20 Breed-specific growth curve fits in White Leghorn laying hen of the Richards (solid), West (dashed), Von Bertalanffy (dotted), Monomolecular (longdash), and Gompertz (dotdash) curves.

# Breed-specific coefficients

Table 3 Coefficients for the breed specific fits for beef cattle.

|  | Male  (kg) | Female  (kg) | Ref | Rich_d_ | | | | West_breed_ | | | Bert_breed_ | | | Mono_breed_ | | | Gomp_breed_ | | | Rich_breed_ | | | |  |
| --- | --- | --- | --- | --- | --- | --- | --- | --- | --- | --- | --- | --- | --- | --- | --- | --- | --- | --- | --- | --- | --- | --- | --- | --- |
| Breed |  |  |  | A | W_0_ | kU | d | A | W_0_ | kU | A | W_0_ | kU | A | W_0_ | kU | A | W_0_ | kU | A | W_0_ | kU | d | |
| Aberdeen  Angus (F) | 800-900 | 550-750 | [3] | 763.1 | 35.2 | 1 | 0.38 | 637.3 | 43.2 | 1.3 | 656.9 | 41.5 | 1.2 | 1292.6 | 25.7 | 0.7 | 594.3 | 47.9 | 1.4 | 768.1 | 35 | 1 | 0.37 | |
| Australian  Angus (Mix) | - | - | - | 946.8 | 71 | 0.8 | 0.38 | 811.3 | 80.8 | 0.9 | 833.6 | 78.8 | 0.9 | 1373.4 | 58.3 | 0.7 | 760.4 | 86.4 | 1 | 1237.2 | 64 | 0.7 | 0.09 | |
| Friesian (M) | 1150-1200 | 650-750 | [3] | 1158.4 | 29.5 | 0.9 | 0.38 | 788.5 | 35.9 | 1.3 | 835.4 | 34.5 | 1.3 | 1760.6 | 25.2 | 0.7 | 695.5 | 39.8 | 1.6 | 1017.7 | 31.3 | 1 | 0.47 | |
|  |  |  |  |  |  |  |  |  |  |  |  |  |  |  |  |  |  |  |  |  |  |  |  | |
| Hereford (M) | 820-1147 | 500-800 | [3] | 1183.2 | 32.9 | 0.9 | 0.38 | 963 | 48.2 | 1.1 | 995.1 | 44.8 | 1 | 2724.9 | 21 | 0.4 | 894.8 | 57.5 | 1.2 | 1189.4 | 32.7 | 0.8 | 0.38 | |
| Holstein  Friesian (M) | 1100-1500 | 650-800 | [3] | 6325.2 | 24.3 | 0.2 | 0.38 | 1349.1 | 29.9 | 1 | 1570.2 | 28.7 | 0.8 | 4321.5 | 25.3 | 0.2 | 1012.5 | 33.4 | 1.3 | 2662.4 | 26.2 | 0.5 | 0.49 | |
| Japanese  Black (Mix/F) | 809 | 512 | [4] | 540 | 0.7 | 2.1 | 0.38 | 522.6 | 15.9 | 2.1 | 525.7 | 12.6 | 2.1 | 577.8 | -43.1 | 2.6 | 515 | 24.8 | 2.1 | 534.1 | 5.2 | 2.1 | 0.49 | |
| Limousin  Friesian (M) | 980-1150 | 670-750 | [3] Limousin | 1354.8 | 23.5 | 0.8 | 0.38 | 833.6 | 29.8 | 1.3 | 893.3 | 28.4 | 1.2 | 1963.7 | 24.2 | 0.6 | 720 | 33.6 | 1.5 | 1030 | 26.8 | 1 | 0.54 | |
| Piemont  Friesian (M) | 950 | 600 | [3] Piemont | 1213.6 | 36.3 | 0.9 | 0.38 | 830.4 | 42 | 1.3 | 879.4 | 40.7 | 1.3 | 1796.6 | 27.4 | 0.7 | 733.1 | 45.7 | 1.6 | 964.7 | 39.8 | 1.1 | 0.56 | |
| Simbrasil (Mix) | - | 500-700 | [4] | 816.1 | 53.4 | 0.9 | 0.38 | 700.2 | 60.9 | 1.1 | 718.8 | 59.3 | 1 | 1225.2 | 45.2 | 0.7 | 658.6 | 65.4 | 1.1 | 977.4 | 50.1 | 0.8 | 0.17 | |
| Simmental (Mix) | 900-1280 | 500-815 | [3] | 859.5 | 60.1 | 0.9 | 0.38 | 739.6 | 70.7 | 1 | 759.7 | 68.6 | 1 | 1193.7 | 43.7 | 0.8 | 693 | 76.4 | 1.1 | 921.8 | 56 | 0.8 | 0.27 | |

A: mature weight (kg), W_0_: birth weight (kg), kU: maximal relative growth rate (10^-3^/day), d: Richards curve shape parameter. Sex is indicated next to each breed as male (M), female (F) or mixed sex (Mix). Literature values of male and female adult weights are provided, if available.

Table 4 Coefficients for the breed specific fits for female dairy cattle.

|  | Female  (kg) | Ref | Rich_d_ | | | | West_breed_ | | | Bert_breed_ | | | Mono_breed_ | | | Gomp_breed_ | | | Rich_breed_ | | | |  |
| --- | --- | --- | --- | --- | --- | --- | --- | --- | --- | --- | --- | --- | --- | --- | --- | --- | --- | --- | --- | --- | --- | --- | --- |
| Breed |  |  | A | W_0_ | kU | d | A | W_0_ | kU | A | W_0_ | kU | A | W_0_ | kU | A | W_0_ | kU | A | W_0_ | kU | d | |
| Ayrshire | 540-590 | [3] | 734.8 | 50.5 | 0.93 | 0.58 | 684.9 | 54.1 | 1 | 707.7 | 52.3 | 1 | 1396.9 | 33.9 | 0.6 | 634.5 | 58.8 | 1.1 | 963.2 | 41.1 | 0.7 | 0.22 | |
| Brown  Swiss | 590-640 | [4] | 750.8 | 50.4 | 1.03 | 0.58 | 707.5 | 54.6 | 1.1 | 727.3 | 52.6 | 1.1 | 1234.4 | 30.2 | 0.7 | 662.6 | 60.2 | 1.2 | 874.7 | 42 | 0.9 | 0.30 | |
| Guernsey | 450-500 | [3] | 692.7 | 43.5 | 0.99 | 0.58 | 646 | 47 | 1.1 | 667.3 | 45.3 | 1 | 1318.2 | 26.8 | 0.6 | 598.9 | 51.6 | 1.2 | 686 | 44 | 1 | 0.60 | |
| Holstein | 650-800 | [3] Holstein Friesian | 752.7 | 45.6 | 1.10 | 0.58 | 704.9 | 49.3 | 1.2 | 726.7 | 47.5 | 1.1 | 1384.4 | 28.9 | 0.7 | 656.7 | 54.4 | 1.3 | 707.9 | 49.1 | 1.2 | 0.74 | |
| Holstein  Friesian | 650-800 | [3] Holstein Friesian | 675.6 | 36.0 | 1.27 | 0.58 | 645.2 | 39.1 | 1.3 | 659.1 | 37.6 | 1.3 | 1015.6 | 27 | 1 | 614.3 | 43.6 | 1.4 | 601.1 | 46.1 | 1.5 | 1.15 | |
| Jersey | 360-550 | [4] | NA | NA | NA | NA | 473.6 | 35.1 | 1.1 | 487.9 | 33.8 | 1.1 | 883.8 | 19 | 0.7 | 441.8 | 38.8 | 1.2 | 621.9 | 25.7 | 0.9 | 0.25 | |
| Milking  Shorthorn | 500-700 | [3] | 747.8 | 46.1 | 1.00 | 0.58 | 700.2 | 50.2 | 1.1 | 721.9 | 48.2 | 1 | 1340.1 | 26.6 | 0.6 | 651.6 | 55.4 | 1.2 | 798.4 | 42.7 | 0.9 | 0.46 | |

A: mature weight (kg), W_0_: birth weight (kg), kU: maximal relative growth rate (10^-3^/day), d: Richards curve shape parameter. Rich_d_ for Jersey was not computed as it was held separately from other breed data. Literature values of male and female adult weights are provided, if available.

Table 5 Coefficients for the breed specific fits for laying hens.

|  | Rich_d_ | | | | West_breed_ | | | Bert_breed_ | | | Mono_breed_ | | | Gomp_breed_ | | | Rich_breed_ | | | |
| --- | --- | --- | --- | --- | --- | --- | --- | --- | --- | --- | --- | --- | --- | --- | --- | --- | --- | --- | --- | --- |
| Breed | A | W_0_ | kU | d | A | W_0_ | kU | A | W_0_ | kU | A | W_0_ | kU | A | W_0_ | kU | A | W_0_ | kU | d |
| Azur | 1.76 | 0.07 | 7.0 | 0.99 | 1.77 | 0.04 | 7.2 | 1.77 | 0.03 | 7.3 | 1.79 | -0.27 | 11.4 | 1.76 | 0.07 | 7 | 1.76 | 0.05 | 7.2 | 0.80 |
| Babcock Brown | 1.96 | 0.06 | 7.4 | 0.99 | 1.96 | 0.02 | 7.7 | 1.97 | 0.01 | 7.8 | 1.99 | -0.35 | 11.7 | 1.96 | 0.06 | 7.4 | 1.96 | 0.03 | 7.6 | 0.79 |
| Babcock White | 1.74 | 0.06 | 7.5 | 0.99 | 1.74 | 0.03 | 7.7 | 1.74 | 0.01 | 7.9 | 1.76 | -0.3 | 12.1 | 1.74 | 0.06 | 7.5 | 1.74 | 0.07 | 7.4 | 1.09 |
| Bovans Black | 2.21 | 0.05 | 7.3 | 0.99 | 2.21 | 0.01 | 7.6 | 2.21 | 0 | 7.7 | 2.24 | -0.47 | 11.4 | 2.21 | 0.05 | 7.3 | 2.21 | 0.1 | 7 | 1.40 |
| Bovans Brown | 1.92 | 0.05 | 7.7 | 0.99 | 1.92 | 0.02 | 8 | 1.92 | 0.01 | 8.1 | 1.95 | -0.37 | 11.9 | 1.92 | 0.05 | 7.7 | 1.92 | 0.04 | 7.8 | 0.95 |
| Bovans White | 1.69 | 0.06 | 7.4 | 0.99 | 1.69 | 0.03 | 7.6 | 1.69 | 0.02 | 7.8 | 1.71 | -0.29 | 11.9 | 1.69 | 0.06 | 7.4 | 1.69 | 0.07 | 7.3 | 1.10 |
| Dekalb Brown | 1.92 | 0.05 | 7.7 | 0.99 | 1.93 | 0.02 | 7.9 | 1.93 | 0.01 | 8 | 1.95 | -0.37 | 11.8 | 1.92 | 0.05 | 7.6 | 1.92 | 0.05 | 7.7 | 0.98 |
| Dekalb White | 1.92 | 0.05 | 7.7 | 0.99 | 1.93 | 0.02 | 7.9 | 1.93 | 0.01 | 8 | 1.95 | -0.37 | 11.8 | 1.92 | 0.05 | 7.6 | 1.92 | 0.05 | 7.7 | 0.98 |
| Hisex Brown | 1.92 | 0.05 | 7.6 | 0.99 | 1.93 | 0.01 | 7.9 | 1.93 | 0 | 8.1 | 1.95 | -0.42 | 12 | 1.92 | 0.05 | 7.6 | 1.92 | 0.05 | 7.6 | 0.99 |
| Hisex White | 1.69 | 0.06 | 7.4 | 0.99 | 1.69 | 0.03 | 7.6 | 1.69 | 0.02 | 7.8 | 1.71 | -0.29 | 11.9 | 1.69 | 0.06 | 7.4 | 1.69 | 0.07 | 7.3 | 1.10 |
| Hy-line W-36 | 1.58 | 0.04 | 8.5 | 0.99 | 1.58 | 0.01 | 8.8 | 1.58 | 0.01 | 8.9 | 1.6 | -0.32 | 13.5 | 1.58 | 0.04 | 8.5 | 1.58 | 0.04 | 8.5 | 0.98 |
| Hy-line W-80 | 1.73 | 0.07 | 7.2 | 0.99 | 1.73 | 0.04 | 7.5 | 1.73 | 0.03 | 7.6 | 1.75 | -0.28 | 12 | 1.73 | 0.08 | 7.2 | 1.73 | 0.04 | 7.5 | 0.76 |
| ISA Brown | 1.93 | 0.05 | 7.7 | 0.99 | 1.93 | 0.02 | 8 | 1.93 | 0.01 | 8.1 | 1.96 | -0.37 | 12 | 1.93 | 0.05 | 7.7 | 1.93 | 0.05 | 7.7 | 1.01 |
| ISA White | 1.74 | 0.06 | 7.5 | 0.99 | 1.74 | 0.03 | 7.7 | 1.74 | 0.01 | 7.9 | 1.76 | -0.3 | 12.1 | 1.74 | 0.06 | 7.5 | 1.74 | 0.07 | 7.4 | 1.09 |
| Lohmann Br. C. | 2.02 | 0.06 | 7.3 | 0.99 | 2.02 | 0.03 | 7.5 | 2.02 | 0.01 | 7.7 | 2.05 | -0.35 | 11.6 | 2.02 | 0.07 | 7.3 | 2.02 | 0.05 | 7.4 | 0.93 |
| Noirans | 2.19 | 0.07 | 7.5 | 0.99 | 2.2 | 0.03 | 7.7 | 2.2 | 0.02 | 7.8 | 2.24 | -0.37 | 11.7 | 2.19 | 0.07 | 7.4 | 2.2 | 0.05 | 7.6 | 0.84 |
| Shaver Black | 2.15 | 0.05 | 7.2 | 0.99 | 2.15 | 0.02 | 7.4 | 2.15 | 0.01 | 7.6 | 2.18 | -0.42 | 11.2 | 2.14 | 0.05 | 7.2 | 2.14 | 0.08 | 7.1 | 1.18 |
| Shaver Brown | 1.92 | 0.05 | 7.7 | 0.99 | 1.93 | 0.02 | 7.9 | 1.93 | 0.01 | 8 | 1.95 | -0.37 | 11.8 | 1.92 | 0.05 | 7.6 | 1.92 | 0.05 | 7.7 | 0.98 |
| Shaver White | 1.69 | 0.06 | 7.4 | 0.99 | 1.69 | 0.03 | 7.6 | 1.69 | 0.02 | 7.8 | 1.71 | -0.29 | 11.9 | 1.69 | 0.06 | 7.4 | 1.69 | 0.07 | 7.3 | 1.10 |
| Warren | 2.07 | 0.07 | 7.6 | 0.99 | 2.07 | 0.03 | 7.9 | 2.08 | 0.02 | 8 | 2.1 | -0.36 | 12.1 | 2.07 | 0.07 | 7.6 | 2.07 | 0.03 | 7.8 | 0.76 |
| White Leghorn | NA | NA | NA | NA | 1.71 | 0.04 | 8.1 | 1.72 | 0.03 | 8.1 | 1.89 | -0.04 | 9.5 | 1.7 | 0.06 | 8.2 | 1.73 | 0.03 | 8.1 | 0.61 |

A: mature weight (kg), W_0_: birth weight (kg), kU: maximal relative growth rate (10^-3^/day), d: Richards curve shape parameter. Rich_d_ for White Leghorn was not computed as it was held separately from other breed data.

Table 6 Coefficients for the breed specific fits for male broiler chicken.

|  | Rich_d_ | | | | West_breed_ | | | Bert_breed_ | | | Gomp_breed_ | | | Rich_breed_ | | | |
| --- | --- | --- | --- | --- | --- | --- | --- | --- | --- | --- | --- | --- | --- | --- | --- | --- | --- |
| Breed | A | W_0_ | kU | d | A | W_0_ | kU | A | W_0_ | kU | A | W_0_ | kU | A | W_0_ | kU | d |
| Arbor Acres Plus | 8.52 | 0.05 | 12.9 | 0.84 | 9.51 | 0.04 | 11.5 | 10.79 | 0.02 | 10.1 | 7.45 | 0.07 | 15 | 8.43 | 0.05 | 13.1 | 0.85 |
| Cobb 500 | 8.15 | 0.05 | 14.3 | 0.84 | 8.89 | 0.03 | 13 | 9.79 | 0.02 | 11.7 | 7.31 | 0.07 | 16.2 | 8.71 | 0.04 | 13.2 | 0.77 |
| Ranger Premium | 5.73 | 0.04 | 12.6 | 0.84 | 6.13 | 0.03 | 11.6 | 6.6 | 0.02 | 10.6 | 5.26 | 0.06 | 14 | 5.64 | 0.04 | 12.8 | 0.87 |
| Ross 308 | 8.63 | 0.05 | 12.6 | 0.84 | 9.24 | 0.03 | 11.6 | 9.95 | 0.02 | 10.7 | 7.91 | 0.08 | 14.1 | 8.63 | 0.05 | 12.6 | 0.84 |
| Ross 708 | 8.6 | 0.05 | 12.3 | 0.84 | 9.25 | 0.03 | 11.3 | 10.03 | 0.02 | 10.3 | 7.84 | 0.08 | 13.8 | 8.53 | 0.05 | 12.4 | 0.86 |
| Rowan 708 | 6.2 | 0.04 | 12.6 | 0.84 | 6.64 | 0.03 | 11.6 | 7.15 | 0.02 | 10.6 | 5.69 | 0.06 | 14 | 6.1 | 0.05 | 12.8 | 0.87 |

A: mature weight (kg), W_0_: birth weight (kg), kU: maximal relative growth rate (10^-3^/day), d: Richards curve shape parameter. The curve Mono_breed_ did not achieve convergence.

Table 7 Coefficients for the breed specific fits for female broiler chicken.

|  | Rich_d_ | | | | West_breed_ | | | Bert_breed_ | | | Gomp_breed_ | | | Rich_breed_ | | | |
| --- | --- | --- | --- | --- | --- | --- | --- | --- | --- | --- | --- | --- | --- | --- | --- | --- | --- |
| Breed | A | W_0_ | kU | d | A | W_0_ | kU | A | W_0_ | kU | A | W_0_ | kU | A | W_0_ | kU | d |
| Arbor Acres Plus | 6.62 | 0.05 | 13.4 | 0.82 | 7.07 | 0.04 | 12.4 | 7.82 | 0.03 | 11.1 | 5.79 | 0.07 | 15.6 | 6.57 | 0.05 | 13.5 | 0.83 |
| Cobb 500 | 7.49 | 0.04 | 13.4 | 0.82 | 8.02 | 0.03 | 12.5 | 8.9 | 0.02 | 11.2 | 6.53 | 0.07 | 15.7 | 7.72 | 0.04 | 13 | 0.79 |
| Ranger Premium | 4.44 | 0.04 | 12.9 | 0.82 | 4.63 | 0.03 | 12.3 | 4.91 | 0.02 | 11.5 | 4.07 | 0.06 | 14.5 | 4.39 | 0.04 | 13.2 | 0.84 |
| Ross 308 | 6.7 | 0.05 | 13 | 0.82 | 6.98 | 0.04 | 12.4 | 7.41 | 0.03 | 11.5 | 6.13 | 0.08 | 14.6 | 6.71 | 0.05 | 13 | 0.81 |
| Ross 708 | 6.7 | 0.05 | 12.5 | 0.82 | 7.01 | 0.04 | 11.9 | 7.49 | 0.03 | 11 | 6.09 | 0.08 | 14.1 | 6.74 | 0.05 | 12.5 | 0.81 |
| Rowan 708 | 4.81 | 0.04 | 12.9 | 0.82 | 5.01 | 0.03 | 12.3 | 5.32 | 0.02 | 11.5 | 4.4 | 0.07 | 14.5 | 4.74 | 0.05 | 13.2 | 0.84 |

A: mature weight (kg), W_0_: birth weight (kg), kU: maximal relative growth rate (10^-3^/day), d: Richards curve shape parameter. The curve Mono_breed_ did not achieve convergence.

Table 8 Coefficients for the breed specific fits for sheep.

|  | Male  (kg) | Female  (kg) | Ref | West_breed_ | | | Bert_breed_ | | | Gomp_breed_ | | |
| --- | --- | --- | --- | --- | --- | --- | --- | --- | --- | --- | --- | --- |
| Breed |  |  |  | A | W_0_ | kU | A | W_0_ | kU | A | W_0_ | kU |
| Improved Valachian (Mix) | 60-70 | 45-50 | [4] | 143.0 | 6.2 | 2.5 | 193.1 | 6.2 | 2.0 | 85.5 | 6.2 | 3.8 |
| Karagouniko (F) | 80 | 57 | [5] | 130.0 | 9.2 | 1.0 | 138.2 | 9.0 | 1.0 | 113.4 | 9.7 | 1.2 |
| Karagouniko (M) | 80 | 57 | [5] | 119.6 | 9.8 | 1.5 | 123.7 | 9.5 | 1.5 | 110.6 | 10.4 | 1.7 |
| Konya Merino (F) | - | 55 | [4] | 57.4 | 6.5 | 2.4 | 58.0 | 6.3 | 2.4 | 55.8 | 7.1 | 2.4 |
| Konya Merino (M) | - | 55 | [4] | 79.5 | 8.3 | 1.9 | 80.9 | 8.1 | 1.9 | 76.1 | 9.0 | 2.0 |
| Lacaune (Mix) | 80-100 | 55-75 | [4] | 114.2 | 5.2 | 3.4 | 145.1 | 5.2 | 2.8 | 74.2 | 5.3 | 5.0 |
| Manchega (F) | 85-90 | 55-60 | [4] | 51.7 | 6.6 | 4.8 | 52.0 | 6.3 | 4.8 | 51.0 | 7.3 | 4.7 |
| Spanish Merino (Mix) | 80-85 | 50-55 | [4] | 55.9 | 4.2 | 4.7 | 61.2 | 4.1 | 4.3 | 46.1 | 4.3 | 5.8 |
| Tsigai (Mix) | 70-80 | 50-55 | [4] | 32.8 | 4.9 | 7.6 | 34.7 | 4.9 | 7.2 | 29.0 | 4.9 | 8.7 |

A: mature weight (kg), W_0_: birth weight (kg), kU: maximal relative growth rate (10^-3^/day), d: Richards curve shape parameter. Sex is indicated next to each breed as male (M), female (F) or mixed sex (Mix). Literature values of male and female adult weights are provided, if available. The Rich_d_, Mono_breed_ and Rich_breed_ models did not achieve convergence for the breed-specific fits.

Table 9 Literature references used in the study which also employ curve comparisons.

| Species | Breed | Curves compared | Ref. |
| --- | --- | --- | --- |
| Beef cattle | Hereford | Brody, **Gompertz**, Logistic, Richards, **Von Bertalanffy** | [6] |
| Beef cattle | Holstein-Friesian | Gompertz, **Richards**, Logistic, Von Bertalanffy | [7] |
| Sheep | Konya Merino | **Quadratic polynomial**, Cubic polynomial, **Gompertz**, Logistic | [8] |

*In bold the preferred curve model by the authors of the referred studies.*

# References

[1] J. Pinheiro and D. M. Bates, *Mixed-Effects Models in S and S-PLUS*. Springer New York, NY, 2000. [Online]. Available: https://doi.org/10.1007/b98882

[2] J. C. Pinheiro, “Model building using covariates in nonlinear mixed-effects models,” *Journal de la Société française de statistique*, vol. 143, no. 1–2, pp. 79–101, 2002.

[3] *CABI*. in CABI Compendium. Wallingford UK: CAB International, 2025. [Online]. Available: https://www.cabidigitallibrary.org/

[4] “Mason’s world encyclopedia of livestock breeds and breeding. Volume 1 and Volume 2,” CABI Books. Accessed: Jun. 24, 2025. [Online]. Available: https://www.cabidigitallibrary.org/doi/book/10.1079/9781845934668.0000

[5] “Amalthia - KARAGOUNIKO.” Accessed: Jun. 24, 2025. [Online]. Available: https://www.amalthia.org/en/breeds/sheep/176-karagouniko/184-karagouniko

[6] A. Mazzini, M. Augusto, L. Aquino, and F. Silva, “Growth curve analysis for Hereford cattle males,” *Ciência e Agrotecnologia*, vol. 27, pp. 1105–1112, Oct. 2003, doi: 10.1590/S1413-70542003000500019.

[7] M. Tutkun, “Growth curve prediction of holstein-fresian bulls using different non-linear model functions,” *Applied Ecology and Environmental Research*, vol. 17, pp. 4409–4416, Jan. 2019, doi: 10.15666/aeer/1702_44094416.

[8] İ. Keskin, B. Dağ, V. Sariyel, and M. Gökmen, “Estimation of growth curve parameters in Konya Merino sheep,” *South African Journal Of Animal Science*, vol. 39, pp. 163–168, Jul. 2009, doi: 10.4314/sajas.v39i2.44390.
